# IDH3 mediates apoptosis of alveolar epithelial cells type 2 due to mitochondrial Ca^2+^ uptake during hypocapnia

**DOI:** 10.1038/cddis.2017.403

**Published:** 2017-08-24

**Authors:** Martina Kiefmann, Sascha Tank, Paula Keller, Christian Börnchen, Jan L Rinnenthal, Marc-Oliver Tritt, Leonie Schulte-Uentrop, Cynthia Olotu, Alwin E Goetz, Rainer Kiefmann

**Affiliations:** 1 Department of Anesthesiology, University Hospital Hamburg-Eppendorf, Martinistrasse 52, Hamburg 20251, Germany; 2 Department of Neuropathology, Charite – Universitätsmedizin Charitéplatz 1 | Virchowweg 15, Berlin 10117, Germany

## Abstract

In adult respiratory distress syndrome (ARDS) pulmonary perfusion failure increases physiologic dead-space (V_D_/V_T_) correlating with mortality. High V_D_/V_T_ results in alveolar hypocapnia, which has been demonstrated to cause edema formation, atelectasis, and surfactant depletion, evoked, at least in part, by apoptosis of alveolar epithelial cells (AEC). However, the mechanism underlying the hypocapnia-induced AEC apoptosis is unknown. Here, using fluorescent live-cell imaging of cultured AEC type 2 we could show that in terms of CO_2_ sensing the tricarboxylic acid cycle enzyme isocitrate dehydrogenase (IDH) 3 seems to be an important player because hypocapnia resulted independently from pH in an elevation of IDH3 activity and subsequently in an increase of NADH, the substrate of the respiratory chain. As a consequence, the mitochondrial transmembrane potential (ΔΨ) rose causing a Ca^2+^ shift from cytosol into mitochondria, whereas the IDH3 knockdown inhibited these responses. Furthermore, the hypocapnia-induced mitochondrial Ca^2+^ uptake resulted in reactive oxygen species (ROS) production, and both the mitochondrial Ca^2+^ uptake and ROS production induced apoptosis. Accordingly, we provide evidence that in AEC type 2 hypocapnia induces elevation of IDH3 activity leading to apoptosis. This finding might give new insight into the pathogenesis of ARDS and may help to develop novel strategies to reduce tissue injury in ARDS.

The adult respiratory distress syndrome (ARDS) is a typical complication of ventilated intensive care patients with an incidence of almost 10% and a mortality rate of ~40%. There are many predisposing factors known, including pneumonia, sepsis, or trauma. Inflammation, alveolar-capillary barrier dysfunction, development of lung edema, and formation of atelectasis typically in the dorsal regions of both lungs are the hallmarks of ARDS. Accordingly, opening of atelectatic lung regions, avoiding their re-collapse by applying a positive end-expiratory pressure, reducing the tidal volume during ventilation, and, if necessary, extracorporeal oxygenation and CO_2_ removal are the treatment approaches in ARDS.

Although the pathogenesis of ARDS has been extensively studied over the last decades, a causal therapy has not been found yet. During the last years it became clear that in both the ARDS and its experimental equivalent, the acute lung injury, intravascular coagulation, and perfusion disorders lead to an increase of the physiologic dead-space fraction (V_D_/V_T_), which is associated with increased mortality.^1^ In contrast to the CO_2_ accumulation in the arterial blood, the CO_2_ concentration in alveoli may drop, especially in lung regions with high V_D_/V_T_. It has been shown that alveolar hypocapnia may contribute to tissue injury, including depletion of surfactant, which is produced by alveolar epithelial cells (AEC) type 2 and normally opposes the alveolar-collapsing tendency by lowering the air–liquid surface tension. Furthermore, reduced secretion of surfactant is commonly seen in ARDS patients and is associated with worse outcomes especially in critically-ill patients (which has been excellently reviewed by J Laffey *et al.*^2, 3, 4, 5, 6^). Thus, the alveolar hypocapnia-induced AEC type 2 damage seems to be likely in ARDS. However, the underlying mechanism has not been elucidated yet.

Apoptosis of AEC potentially has an important role in ARDS. Decreased size and condensation of the chromatin of AEC type 2 have been shown early in human ARDS.^7^ Moreover, caspase-cleaved cytokeratin-18, a marker for epithelial cell apoptosis, increases in bronchoalveolar lavage fluids during ARDS.^8^

Several pathways of apoptosis induction are known, also in the context of ARDS. One important trigger for apoptosis is the mitochondrial Ca^2+^ overload, especially in combination with increased mitochondrial production of reactive oxygen species (ROS).^9^ Importantly, mitochondrial Ca^2+^ uptake and ROS production can mutually be dependent. The driving force for mitochondrial Ca^2+^ uptake is the negative transmembrane potential gradient (ΔΨ) across the mitochondrial inner membrane.^10, 11^ There are reports that ΔΨ can be increased by stimulation of the respiratory chain with its substrate NADH, which is produced in the tricarboxylic acid (TCA) cycle by the enzymes isocytrate dehydrogenase (IDH) 3 and *α*-ketoglutarate dehydrogenase^12, 13, 14, 15^ together with CO_2_.

According to the law of mass action the accumulation of the product may inhibit, whereas a rapid clearance of the product may accelerate its enzymatic reaction rate. We therefore hypothesize that a decrease of the alveolar partial pressure of CO_2_ (pCO_2_), which occurs in lung regions with high V_D_/V_T_, increases IDH3 activity, NADH production, and thus ΔΨ in AEC.

Using real-time fluorescence microscopy of AEC type 2 we could identify that mitochondrial IDH3 is a key player in CO_2_ sensing. Accordingly, under hypocapnic conditions the resulting increase of IDH3 activity elicits an increase of ΔΨ and subsequently a shift of Ca^2+^ from the cytosol into mitochondria. In consequence, the hypocapnia-induced mitochondrial Ca^2+^ uptake leads to elevation of mitochondrial ROS production, and thus apoptosis. The understanding of this context may help to develop strategies to reduce AEC type 2 injury, including formation of atelectasis, in ARDS patients with elevated V_D_/V_T_. Furthermore, the opening of atelectasis, a central element of ADRS therapies, should be re-evaluated in non-perfused lung regions to prevent hypocapnic conditions and thus apoptosis of AEC.

## Results

### Hypocapnia increases IDH3 activity and thus NADH production

**Figure 1:**
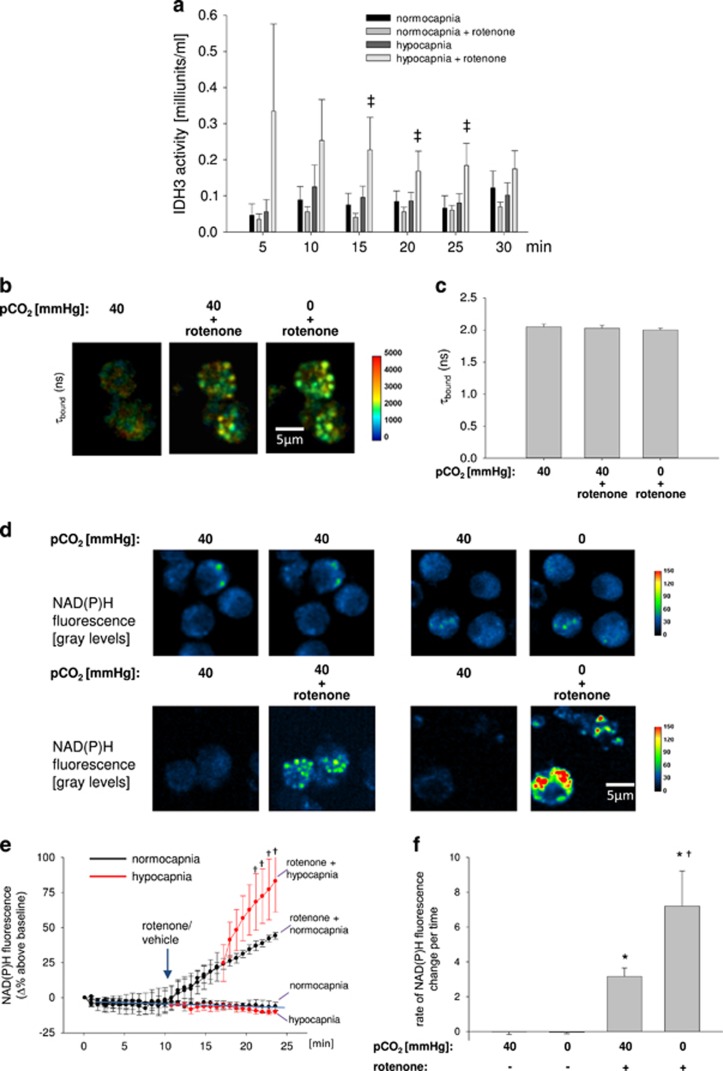

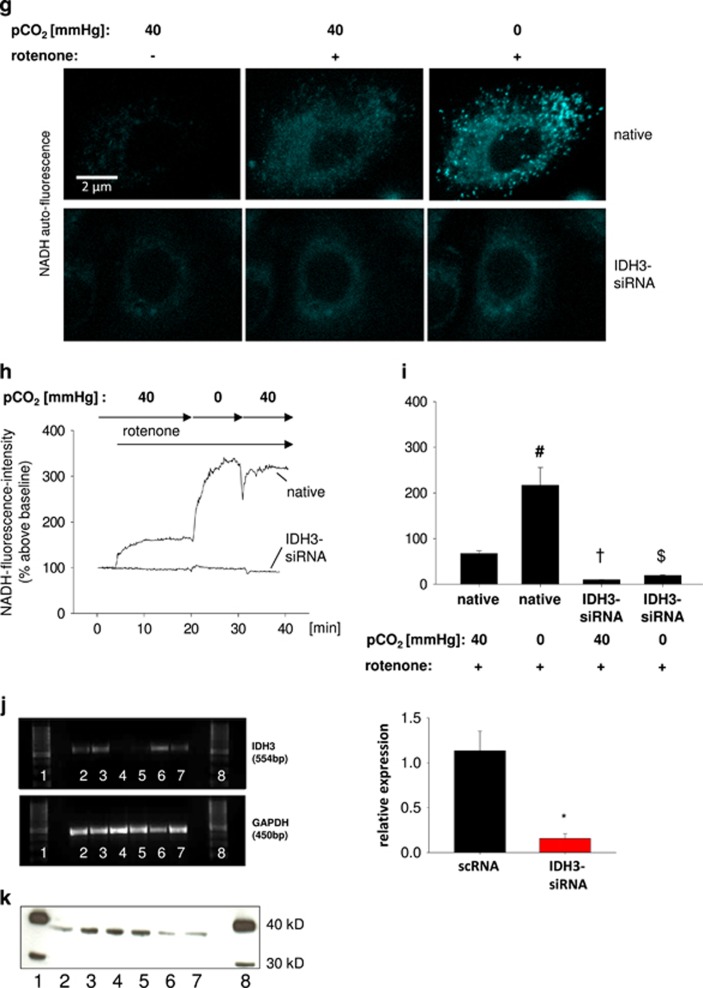
IDH3 activity and NADH concentration in AEC type 2. (**a**) Group data of IDH3 activity in primary isolated AEC type 2 measured every 5 min under normocapnic (pCO_2_: 40 mm Hg) or hypocapnic (pCO_2_: 0 mm Hg) conditions without or with rotenone (1 *μ*M), respectively, at constant extracellular pH of 7.4. 50 000 cells/well. Mean±S.E., ^‡^*P*<0.05 *versus* pCO_2_: 0 mm Hg (hypocapnia) alone. Each measurement of a single experiment was repeated twice. In total, 2–10 cells were analyzed per picture. Each experiment was repeated six times. (**b**) Color-coded images and (**c**) quantification of changes in τ_bound_ in primary isolated AEC type 2 under normocapnic (pCO_2_: 40 mm Hg) conditions alone or in combination with rotenone or after hypocapnia (pCO_2_: 0 mm Hg) in combination with rotenone. Mean±S.E., repeated four times. (**d**) Images of primary isolated AEC type 2 show the pseudocolor-coded NAD(P)H. Upper panel, left group: left picture – normocapnic condition (baseline), right picture – normocapnic condition (control intervention); upper panel, right group: left picture – normocapnic condition (baseline), right picture – hypocapnic condition alone (intervention); lower panel, left group: left picture – normocapnic condition (baseline), right picture – normocapnic condition+rotenonre (intervention); lower panel, right group: left picture – normocapnic condition (baseline), right picture – hypocapnic condition+rotenone (intervention). pH was constant at 7.4. A total of 2–10 cells were analyzed per picture. Experiments were repeated four times. (**e**) Δ percentage change of the NAD(P)H fluorescence in primary isolated AEC type 2 under normocapnic baseline conditions, after rotenone (1 *μ*M) or vehicle application, and again under normo- or hypocapnic conditions, as indicated. Black line represents normocapnic condition, red line represents hypocapnic condition. Experiments were conducted at constant pH of 7.4 throughout. Mean±S.E., ^†^*P*<0.05 *versus* rotenone at pCO_2_ of 40 mm Hg (normocapnia). In total, 2–10 cells were analyzed per image. Experiments were repeated four times. (**f**) Rate of NAD(P)H fluorescence change during baseline conditions, hypocapnia alone, normocapnia in combination with rotenone or hypocapnia in combination with rotenone at constant pH of 7.4, as indicated. Mean±S.E., *P<0.05 *versus* pCO_2_ of 40 mm Hg alone (control), ^†^*P*<0.05 *versus* rotenone at pCO_2_ of 40 mm Hg (normocapnia). In total, 3–10 cells were analyzed per image. Experiments were repeated four times. (**g**) Images showing NADH auto-fluorescence (measured at ex.: 360 nm and em: 510 nm) of native or IDH3-siRNA-treated A549 cells superfused with normocapnic (pCO_2_: 40 mm Hg) or hypocapnic (pCO_2_: 0 mm Hg) buffer without or with rotenone, as indicated. (**h**) Tracing of the percentage change of the NADH auto-fluorescence in native or IDH3-siRNA-treated A549 cells superfused with normo- or hypocapnic buffer, as indicated, at constant pH of 7.4. Rotenone (1 *μ*M) was applicated as indicated. (**i**) Group data of maximal NADH auto-fluorescence response 10 min following rotenone (1 *μ*M) under normocapnic conditions or 10 min following additionally switching to hypocapnic conditions in native or IDH3-siRNA-treated A549 cells in comparison with normocapnic baseline conditions. Experiments were performed at constant extracellular pH of 7.4. Mean±S.E., ^#^*P*<0.05 *versus* baseline (pCO_2_: 40 mm Hg). ^†^*P*<0.05 *versus* rotenone at pCO_2_ of 40 mm Hg in native cells, ^$^*P*<0.05 *versus* rotenone at pCO_2_ of 0 mm Hg in native cells. In total, 4–11 cells were analyzed per picture. Experiments were repeated six times. (**j**) (Left) Gels show RT-PCR products for IDH3- and GAPDH-mRNA in native (lane 2 and 3), in IDH3-siRNA- (lane 4 and 5), and scRNA-treated (lane 6 and 7) A549 cells. Lane 1 and 8: molecular weight ladder. (Right) Group data of relative IDH3-mRNA expression, *P<0.05 *versus* scRNA (control), *n*=4–5 each. (**k**) Western blot show IDH3 protein in native (lane 2 and 3), in scRNA- (lane 4 and 5), and IDH3-siRNA-treated (lane 6 and 7) A549 cells. Lane 1 and 8: molecular weight ladder

Using extracts of primary isolated AEC type 2 we were able to show that hypocapnia at constant pH of 7.4 did not induce a detectible increase of IDH3 activity (Figure 1a). We assumed that the IDH3 product NADH, which was measured for IDH3 activity determination, was oxidized by the complex 1 of the respiratory chain before its colorimetric assay detection. Therefore, we inhibited the complex 1 with rotenone. Under these conditions, hypocapnia-induced NADH production was detectable. Furthermore, the respective NADH production rate was higher in comparison with rotenone alone (Figure 1a). Importantly, the used biochemical assay was specific for the mitochondrial isoenzyme IDH3, because the added co-substrate NAD^+^ does not react with the non-mitochondrial IDH isoforms 1 and 2.

In addition, using biexponential 2-photon fluorescence lifetime imaging microscopy (FLIM) we could show that lifetimes for free (τ_free_) or bound (τ_bound_) NAD(P)H were 438±47 or 2156±295 ps, respectively (Supplementary Figure 1a). In total, 91.8±0.5% of the detected NAD(P)H was bound (Supplementary Figure 1b). According to Blacker *et al.*^16^ τbound measurements can be used to discriminate between NADH and NADPH. These authors predicted that τ_bound_ values for NADH and NADPH were 1.5 and 4.4 ns, respectively. Hence, the measured τ_bound_ values of ~2 indicated that under both normo- and hypocapnic conditions primary isolated AEC type 2 produced rather NADH than NADPH (Figures 1b and c). We could detect the NAD(P)H fluorescence predominantly in small granules located around the non-fluorescent nucleus, which are ascribed to mitochondria,^17^ (Figure 1a Supplementary Figure 1b and d) indicating that under our experimental condition the main NADH production site is the TCA cycle. In line with the enzymatic IDH3 activity assay results, hypocapnia alone did not change NAD(P)H fluorescence in comparison with normocapnic conditions (Figures 1e and f). However, after rotenone pretreatment NAD(P)H fluorescence steadily increased and further increased after additional hypocapnia (Figures 1d–f) in comparison with hypocapnia alone. Because after rotenone alone or in combination with hypocapnia NAD(P)H fluorescence increased, whereas after both interventions τ_bound_ stayed stable at 2 ns we assume that under both conditions, rather NADH than NADPH was produced (Figures 1b and c). All experiments were conducted at constant pH of 7.4.

In accordance with the FLIM experiments on primary isolated AEC type 2, in rotenone-treated A549 cells the NAD(P)H auto-fluorescence increase was higher under hypo- than normocapnic conditions (Figures 1g–i). Importantly, the NAD(P)H auto-fluorescence in A549 cells was confined to mitochondrial localization, indicating again the mitochondrial origin of the NAD(P)H production (Figure 1g). As auto-fluorescence is not specific to NADH we further measured NADH concentration in A549 cells extracts by using an enzymatic assay. We revealed that NADH concentration 15 min upon onset of hypocapnia was only slightly increased in comparison to normocapnia (Supplementary Figure 2). However, after rotenone hypocapnia doubled the NADH concentration within 30 min (Supplementary Figure 2), indicating again that without rotenone treatment the NADH produced under hypocapnia is immediately consumed by the complex 1 of the respiratory chain.

To further elucidate the specificity of IDH3 in the hypocapnia-induced NADH production process we downregulated this enzyme in A549 cells. The mitochondrial IDH3 knockdown blocked the hypocapnia-induced increase of the NAD(P)H auto-fluorescence (Figures 1g–i). The IDH3 knockdown was verified on mRNA and protein level (Figures 1j and k, respectively). Taken together, we conclude first that, independent from pH, hypocapnia increases the mitochondrial IDH3 activity and thus the NADH production rate and second that, directly after its synthesis, NADH is totally consumed by the complex 1 of the respiratory chain.

### The hypocapnia-induced acceleration of IDH3 activity and thus NADH production subsequently increases mitochondrial ΔΨ

**Figure 2:**
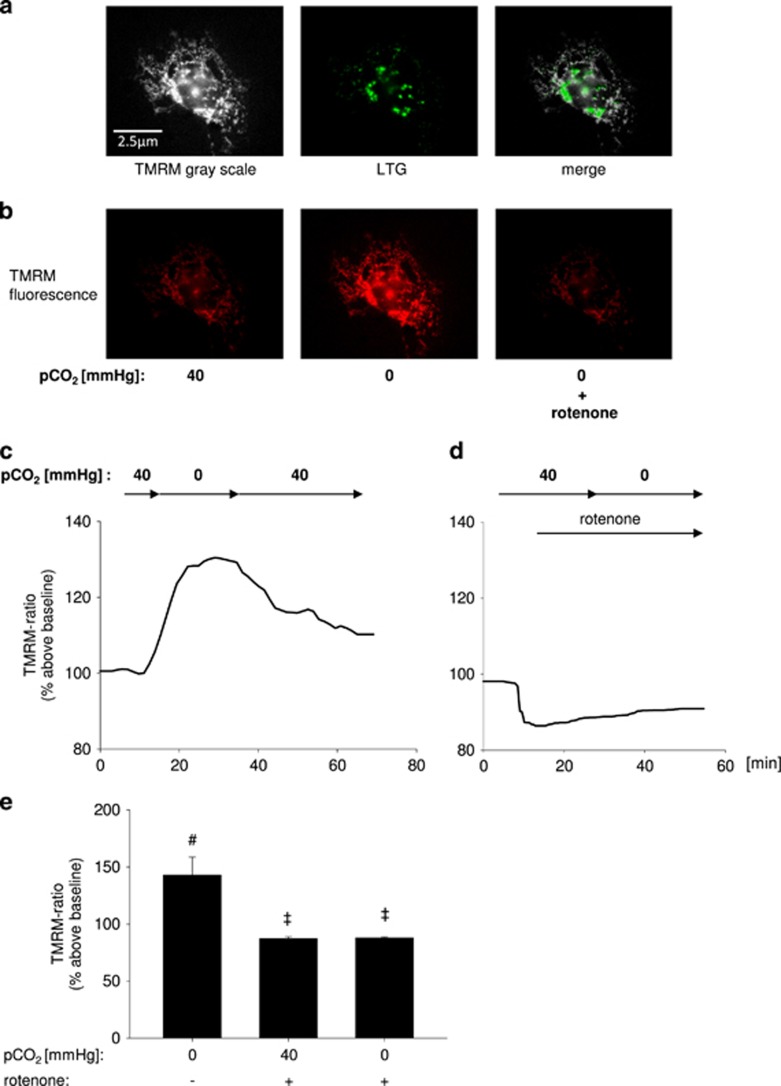

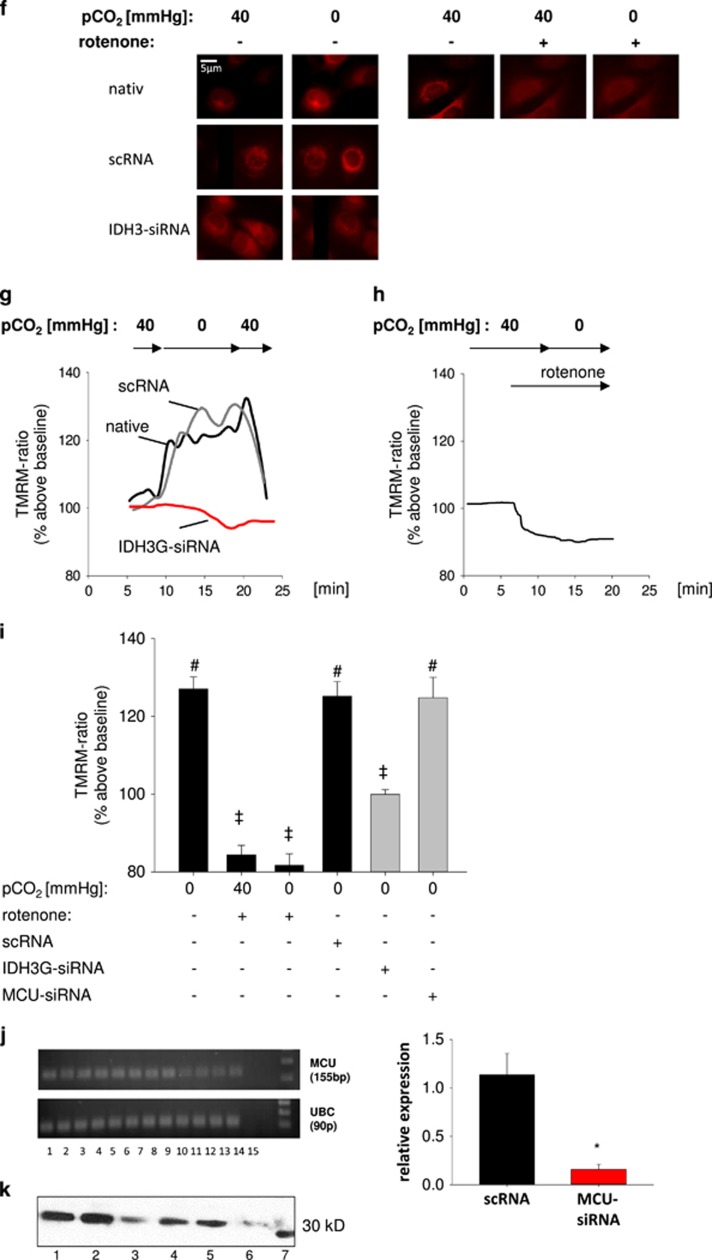
ΔΨ in alveolar epithelial cells *in vitro.* (**a**) Images show primary isolated AEC type 2. For ΔΨ quantification cells were loaded with TMRM. For characterization identical cells were additionally stained with the AEC type 2-specific lamellar body marker LTG (middle). (**b**) Images showing fluorescence of TMRM-loaded primary isolated AEC type 2 superfused with normocapnic (pCO_2_: 40 mm Hg) or hypocapnic (pCO_2_: 0 mm Hg) buffer without or with rotenone (1 *μ*M), as indicated. (**c, d**) Tracing of the ratio between mitochondrial and cytosolic TMRM fluorescence intensity in primary isolated AEC type 2 superfused with normo- or hypocapnic buffer without or with rotenone (1 *μ*M), as indicated, at constant pH of 7.4. (**e**) Group data of maximal TMRM ratio response 15 min after switching from normocapnic (pCO_2_: 40 mm Hg) baseline to hypocapnic (pCO_2_: 0 mm Hg) conditions in primary isolated AEC type 2 at constant extracellular pH of 7.4. Mean±S.E., ^#^*P*<0.05 *versus* baseline (pCO_2_: 40 mm Hg), ^‡^*P*<0.05 *versus* pCO_2_: 0 mm Hg alone. In total, 3–9 cells were analyzed per picture. Experiments were repeated 4–6 times. (**f**) Images show rhod-2 fluorescence of native, scRNA, or IDH3-siRNA-treated A549 cells under normocapnic conditions alone or in combination with rotenone and under hypocapnic condition alone or in combination with rotenone, as indicated. (**g**) Tracing of the ratio between mitochondrial and cytosolic TMRM fluorescence intensity of native, scRNA- or IDH3-siRNA-treated A549 cells superfused with normo- or hypocapnic buffer, as indicated, at constant pH of 7.4. (**h**) Tracing of the ratio between mitochondrial and cytosolic TMRM fluorescence intensity of native A549 cells superfused with normocapnic buffer alone (pCO_2_: 40 mm Hg) or in combination with rotenone, or superfused with hypocapnic buffer (pCO_2_: 0 mm Hg) in combination with rotenone, as indicated, at constant pH of 7.4. (**i**) Group data of maximal TMRM ratio response 15 min after switching from normocapnic (pCO_2_: 40 mm Hg) baseline condition to hypocapnia alone, to normocapnia plus rotenone, or to hypocapnia (pCO_2_: 0 mm Hg) plus rotenone in native, scRNA-, IDH3-siRNA-, or MCU-siRNA-treated A549 cells at constant extracellular pH of 7.4, as indicated. Mean±S.E., ^#^*P*<0.05 *versus* baseline (pCO_2_: 40 mm Hg), ^‡^*P*<0.05 *versus* pCO_2_: 0 mm Hg alone in native or scRNA-treated A549 cells. In total, 4–12 cells were analyzed per picture. Experiments were repeated 4–6 times. (**j**) (Left) Gels show RT-PCR products for MCU- and UBC-mRNA in native (lane 1–4), in scRNA- (lane 5–8), and MCU-siRNA-treated (lane 9–12) A549 cells. Lane 13: no template control, lane 14: negative control, lane 15 molecular weight ladder. (Right) Group data of relative MCU-mRNA expression, *P<0.05 *versus* scRNA (control), *n*=4 each. (**k**) Western blot show MCU protein in native (lane 1 and 4), in scRNA- (lane 2 and 5), and MCU-siRNA-treated (lane 3 and 6) A549 cells. Lane 7: molecular weight ladder

We next determined whether the hypocapnia-induced increase of IDH3 activity, NADH production rate and subsequent respiratory chain NADH consumption elevated ΔΨ. The characterization of the primary isolated AEC type 2 was revealed by the detection of LTG-stained lamellar bodies (Figure 2a). In these cells, hypocapnia at constant pH of 7.4 led to an increase of the ratio between the mitochondrial and cytosolic tetramethylrhodamine methyl ester (TMRM) fluorescence intensity equivalent for an increase of ΔΨ (Figures 2b). Under normocapnic conditions, rotenone decreased, as expected, the TMRM ratio (Figures 2d and e) verifying the correctness of the method. In these rotenone-treated cells, hypocapnia did not increase the TMRM ratio and thus ΔΨ (Figures 2d and e). All these results could be reproduced in A549 cells (Figures 2f–i). Furthermore, in these cells the IDH3 knockdown inhibited the hypocapnia-induced increase of ΔΨ (Figures 2f, g and i). Summarizing, the hypocapnia-induced increase of IDH3 activity, NADH production rate, and subsequent respiratory chain NADH consumption caused an elevation of mitochondrial ΔΨ.

As mentioned before, ΔΨ can be the driving force for mitochondrial Ca^2+^ uptake but it is also well known that mitochondrial Ca^2+^ regulates IDH3 and respiratory chain activity. Accordingly, we conclude that mitochondrial Ca^2+^ is not responsible for the observed ΔΨ changes. We could demonstrate that the silencing of the mitochondrial Ca^2+^ uniporter (MCU) did not inhibit the hypocapnia-induced ΔΨ increase (Figure 2i). The MCU knockdown was verified on mRNA and protein level (Figures 2j and k, respectively).

### Hypocapnia induces mitochondrial Ca^2+^ uptake from the cytosol

**Figure 3:**
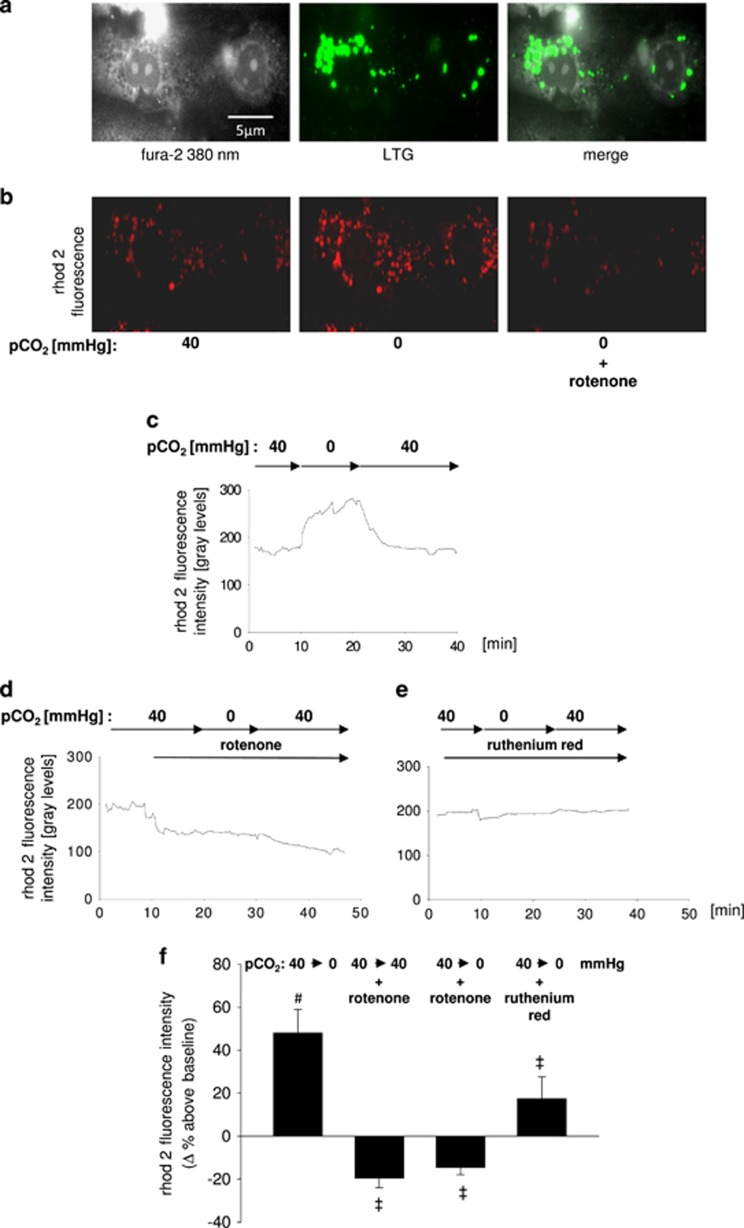

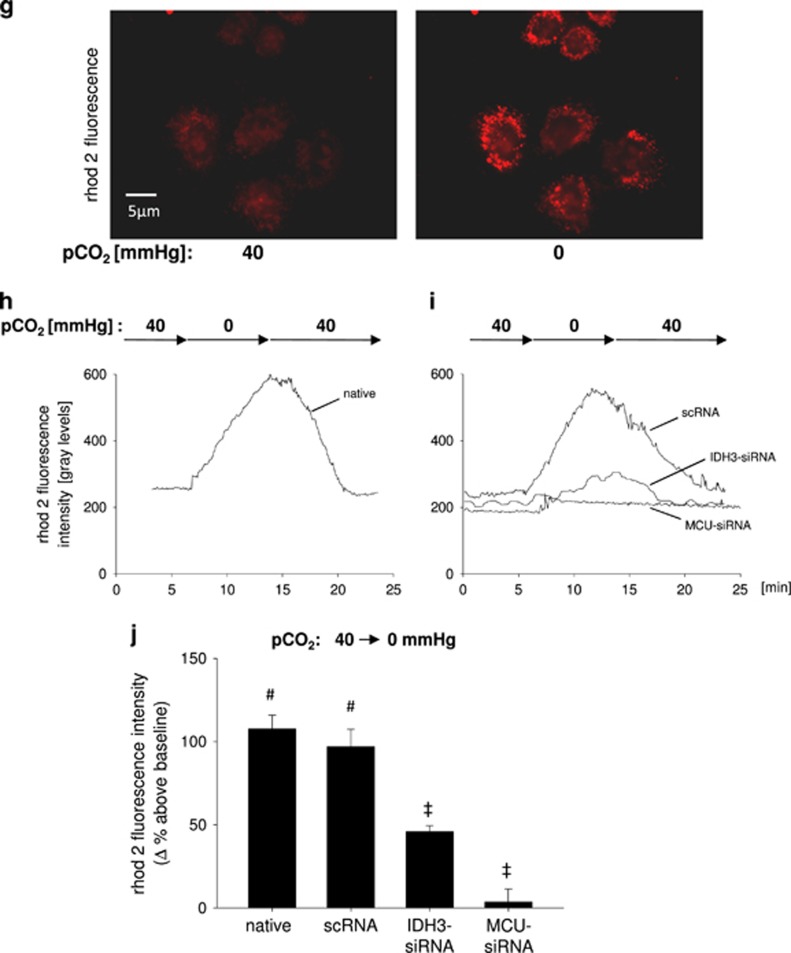
[Ca^2+^]_mito_ in AEC type 2. (**a**) Images show primary isolated AEC type 2. For characterization cells were stained with the cytosolic dye fura 2 and with the AEC type 2-specific lamellar body marker LTG (middle). (**b**) For measurement of [Ca^2+^]_mito,_ AEC type 2 cells were loaded with rhod-2. Images showing fluorescence of rhod-2-loaded primary isolated AEC type 2 under normocapnic (pCO_2_: 40 mm Hg) baseline condition and after hypocapnia (pCO_2_: 0 mm Hg) alone or in combination with rotenone (1 *μ*M), as indicated. (**c–e**) Tracing of rhod-2 fluorescence in primary isolated AEC type 2 superfused with normo- or hypocapnic buffer alone or in combination rotenone (1 *μ*M) or ruthenium red (10 *μ*M), as indicated. Experiments were performed at constant extracellular pH of 7.4, respectively. (**f**) Group data of maximal rhod-2 fluorescence responses in primary isolated AEC type 2 10 min following switching from normocapnic (pCO_2_: 40 mm Hg) baseline to hypocapnic (pCO_2_: 0 mm Hg) conditions without or with rotenone (1 *μ*M) or ruthenium red (10 *μ*M) pretreatment, as indicated. Experiments were performed at constant extracellular pH of 7.4 throughout. Mean±S.E., ^#^*P*<0.05 *versus* baseline (pCO_2_: 40 mm Hg), ^‡^*P*<0.05 *versus* pCO_2_ of 0 mm Hg alone. In total, 5–10 cells were analyzed per picture. Experiments were repeated 6 times. (**g**) Images showing fluorescence of rhod-2-loaded A549 cells under normocapnic (pCO_2_: 40 mm Hg) baseline or hypocapnic (pCO_2_: 0 mm Hg) conditions. (**h, i**) Tracings of rhod-2 fluorescence in native, scRNA-, IDH3-siRNA-, or MCU-siRNA-treated A549 cells. Cells were superfused with normo- or hypocapnic buffer, as indicated. Experiments were performed at constant extracellular pH of 7.4. (**j**) Group data of maximal rhod-2 fluorescence responses in native, scRNA-, IDH3-siRNA-, or MCU-siRNA-treated A549 cells 10 min after switching from normocapnic (pCO_2_: 40 mm Hg) baseline to hypocapnic (pCO_2_: 0 mm Hg) conditions. Experiments were performed at constant extracellular pH of 7.4 throughout. Mean±S.E., ^#^*P*<0.05 *versus* baseline (pCO_2_: 40 mm Hg), ^‡^*P*<0.05 *versus* pCO_2_: 0 mm Hg alone in native or scRNA-transfected cells. 5–10 cells were analyzed per picture. Experiments were repeated 6 times

We next determined in primary isolated AEC type 2 the hypocapnia-induced mitochondrial Ca^2+^ uptake. The characterization of these cells was again revealed by the detection of LTG-stained lamellar bodies (Figure 3a). Switching from normo- to hypocapnic conditions induced a rhod-2 fluorescence intensification, equivalent to an increase of the mitochondrial Ca^2+^ concentration [Ca^2+^]_mito_ (Figures 3b). This response was completely reversible after switching back to normocapnic buffer. All experiments were performed at a constant extracellular pH of 7.4. The mitochondrial localization of the rhod-2 fluorescence was proofed by its co-localization with the mitochondrial marker MitoTracker green (MTG) (data not shown) and by the observation that in AEC type 2 the rhod-2 fluorescence decreases, as expected, after inhibition of the mitochondrial electron chain by rotenone (Figures 3d and f). Furthermore, in primary isolated AEC type 2 cells the hypocapnia-induced increase of the rhod-2 fluorescence intensity could be blocked by either rotenone or the MCU inhibitor ruthenium red (Figures 3d–f). In line with these findings hypocapnia induced an increase of rhod-2 fluorescence in A549 cells at constant pH of 7.4 (Figures 3g). In addition, the hypocapnia-induced mitochondrial Ca^2+^ uptake could be verified by FRET (Supplementary Figure 3A–C). The measurement of mitochondrial Ca^2+^ with FRET was validated by ATP or rotenone as it is well known that ATP increases and rotenone decreases [Ca^2+^]_mito_.^18^ Moreover, in A549 cells the MCU silencing prevented the hypocapnia-induced increase of rhod-2 fluorescence intensity (Figures 3i and j). We therefore assumed that hypocapnia shifts Ca^2+^ from the cytosol into mitochondria. Importantly, IDH3 silencing inhibited the hypocapnia-induced increase of rhod-2 fluorescence intensity indicating that the elevated IDH3 activity and ΔΨ were responsible for the hypocapnia-induced mitochondrial Ca^2+^ uptake (Figures 3i and j).

**Figure 4:**
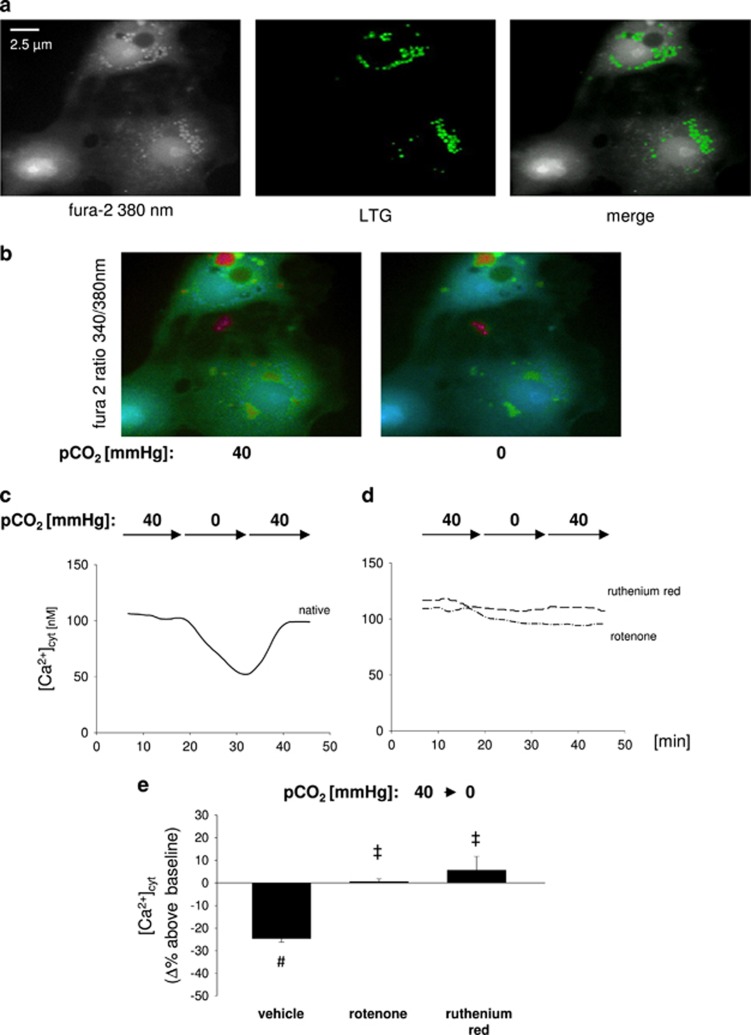

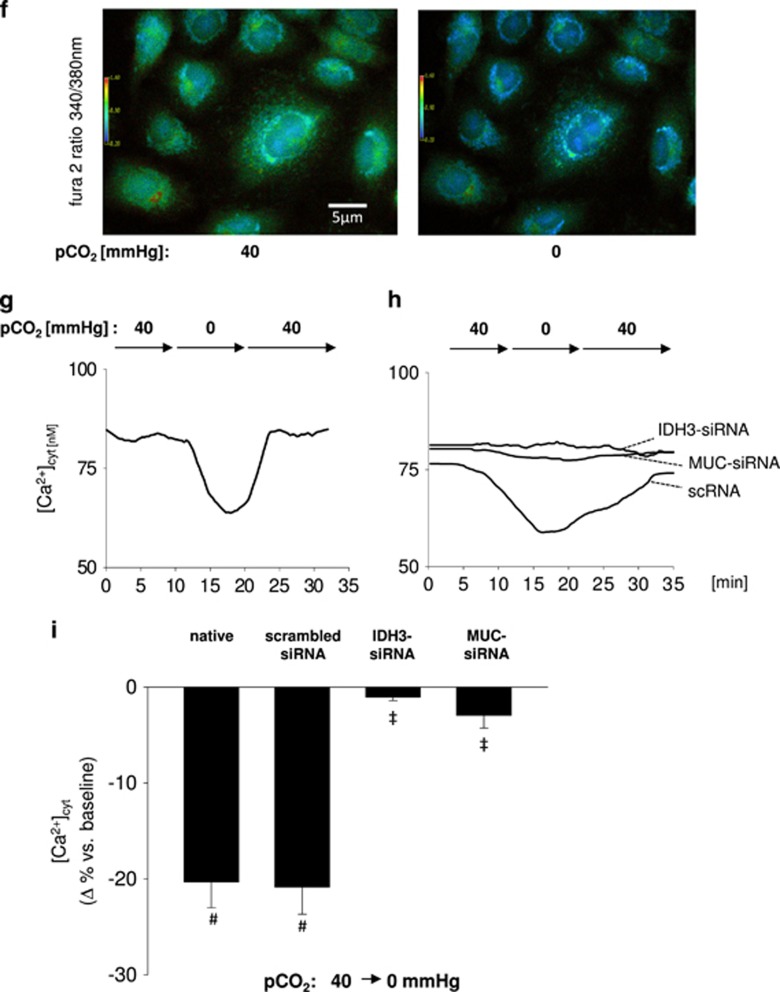
[Ca^2+^]_cyt_, in alveolar epithelial cells *in vitro.* (**a**) Images show primary isolated AEC type 2. For [Ca^2+^]_cyt_ measurement cells were loaded with fura 2 (left). For characterization cells were additionally stained with LTG (middle), a marker for type 2-specific lamellar bodies. (**b**) Images show the 340:380 ratio in pseudocolor of the identical fura 2-loaded primary isolated AEC type 2 under normocapnic (pCO_2_: 40 mm Hg) baseline or hypocapnic (pCO_2_: 0 mm Hg) conditions at constant pH of 7.4. (**c**) Tracing of [Ca^2+^] from an identical epithelial cell superfused with normo- (pCO_2_: 40 mm Hg) or hypocapnic (pCO_2_: 0 mm Hg) buffer, as indicated. (**d**) Tracings of [Ca^2+^] from an identical epithelial cell superfused with normo- (pCO_2_: 40 mm Hg) or hypocapnic (pCO_2_: 0 mm Hg) buffer, as indicated. Cells were pretreated with rotenone or ruthenium red, as indicated. (**e**) Bars show group data of maximal [Ca^2+^]_cyt_ response in primary isolated AEC type 2 15 min after intervention. Cells were pretreated with vehicle, rotenone (1 *μ*M), or ruthenium red (10 *μ*M), as indicated. Under baseline conditions cells were superfused with buffer at following conditions: pCO_2_: 40 mm Hg and pH 7.4 for each group. Buffer conditions after intervention were: pCO_2_: 0 mm Hg and pH 7.4 for each group. Mean±S.E., ^#^*P*<0.05 *versus* baseline (pCO_2_ of 40 mm Hg), ‡ *P*<0.05 *versus* pCO_2_ of 0 mm Hg alone. In total, 3–9 cells were analyzed per picture. Experiments were repeated six times each. (**f**) Images show the pseudocolor-coded 340:380 ratio for fura 2-loaded A549 cells under normocapnic (pCO_2_: 40 mm Hg) baseline or hypocapnic (pCO_2_: 0 mm Hg) conditions at constant pH of 7.4. (**g**) Tracing of [Ca^2+^]_cyt_ from an identical epithelial cell superfused with normo- (pCO_2_: 40 mm Hg) or hypocapnic (pCO_2_: 0 mm Hg) buffer, as indicated. pH was constant at 7.4. (**h**) Tracings of [Ca^2+^]_cyt_ from an identical epithelial cell superfused with normo- (pCO_2_: 40 mm Hg) or hypocapnic (pCO_2_: 0 mm Hg) buffer, as indicated. Cells were untreated or treated with scRNA, IDH3-siRNA, or MCU-siRNA, as indicated. (**i**) Group data of maximal [Ca^2+^]_cyt_ responses 10 min following switching from normocapnic (pCO_2_: 40 mm Hg) baseline to hypocapnic conditions (pCO_2_: 0 mm Hg) at constant extracellular pH of 7.4 in native, scRNA-, IDH3-siRNA-, or MCU-siRNA-treated A549 cells. Mean±S.E., ^#^*P*<0.05 *versus* baseline (pCO_2_: 40 mm Hg), ^‡^*P*<0.05 *versus* pCO_2_: 0 mm Hg in native or scRNA-treated A549 cells. In total, 3–10 cells were analyzed per picture. Experiments were repeated 6 times

To further test if mitochondria incorporate Ca^2+^ from the cytosolic compartment we measured cytosolic Ca^2+^ concentration [Ca^2+^]_cyt_ in primary isolated AEC type 2. A switch of pCO_2_ from 40 to 0 mm Hg decreased [Ca^2+^]_cyt_ by 25±5% *versus* baseline conditions within 10 min in a reversible manner (Figures 4a–c, e). Exposing primary isolated cells to hypocapnic conditions for 30 min revealed that the hypocapnia-induced [Ca^2+^]_cyt_ decrease is transient (Supplementary figure 4). Interestingly, switching back to normocapnic conditions causes an overshooting increase of [Ca^2+^]_cyt_ assuming that cells compensate the hypocapnia-induced [Ca^2+^]_cyt_ decrease by mechanisms, as e.g., decrease of cell membrane Ca^2+^ pump activity. The pretreatment of AEC type 2 cells with ruthenium red or rotenone inhibited the hypocapnia-induced [Ca^2+^]_cyt_ decrease (Figures 4d and e). In accordance to these findings, in A549 cells hypocapnia reduced [Ca^2+^]_cyt_ 20±2%, which was inhibited by rotenone (Figures 4f). In addition, both the MCU and IDH3 silencing inhibited the hypocapnia-induced decrease of [Ca^2+^]_cyt_ (Figure 4h).

We could demonstrate that in native A549 cells ATP alone caused an increase of [Ca^2+^]_cyt_, which was comparable to that after ATP in combination with rotenone. Further, the ATP response in native A549 cells did not differ from the ATP responses in scRNA- or IDH3-siRNA-treated cells. This indicates that both rotenone and IDH3 knockdown did not affect cell viability at least in terms of receptor-mediated Ca^2+^-signaling cascades (Supplementary figure 5).

Furthermore, we were also interested whether pH may play a role in Ca^2+^ response induced by hypocapnia. Without additional buffering, the removing of the dissolved CO_2_ increased the pH of the superfusion buffer from 7.4 to 7.8. Under these conditions hypocapnia induced a similar reduction of [Ca^2+^]_cyt_ in A549 cells as at constant extracellular pH of 7.4 revealed by buffering (Supplementary figure 6A). In contrast, an alkalosis of 7.8 under normocapnic conditions did not induce an alteration of [Ca^2+^]_cyt_ (Supplementary figure 6A). Furthermore, the cytosolic pH was not affected in unbuffered cells (Supplementary figure 6B). We therefore conclude that the hypocapnia-induced [Ca^2+^]_cyt_ responses were pH independent.

Since Ca^2+^ can be shifted directly from the endoplasmatic reticulum (ER) to mitochondria, we measured Ca^2+^ in the ER [Ca^2+^]_ER_. In contrast to [Ca^2+^]_cyt_ and [Ca^2+^]_mito_, hypocapnia did not change [Ca^2+^]_ER_ (Supplementary figure 7A–C). However, this ratio was clearly elevated after ATP application indicating the correctness of the method (Supplementary figure 7A–C). Taken these results together, we conclude that hypocapnia and thus acceleration of IDH3 activity induces a Ca^2+^ shift from the cytosol to the mitochondria, whereas, the ER seems to have a minor role in this scenario.

### Both, the hypocapnia-induced increase of [Ca^2+^]_mito_ and associated mitochondrial ROS production evoke apoptosis of AEC type 2

**Figure 5:**
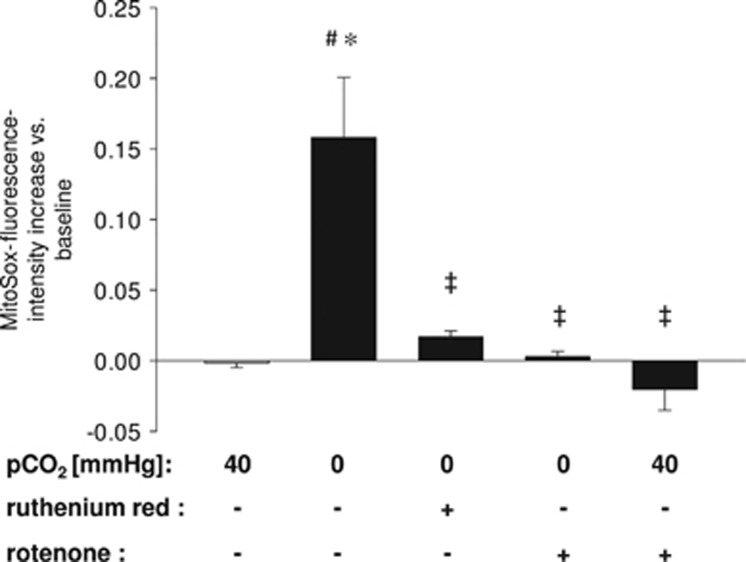
Group data of Mitosox fluorescence increase *versus* normocapnic (pCO_2_: 40 mm Hg) baseline conditions in primary isolated AEC type 2, 20 min following normocapnia (pCO_2_: 40 mm Hg) alone, hypocapnia (pCO_2_: 0 mm Hg) alone or in combination with ruthenium red (10 *μ*M) or rotenone (1 *μ*M), or normocapnia in combination with rotenone (1 *μ*M), as indicated. Experiments were performed at constant extracellular pH of 7.4. Mean±S.E., ^#^*P*<0.05 *versus* baseline (pCO_2_: 40 mm Hg), ^‡^*P*<0.05 *versus* pCO_2_: 0 mm Hg alone, *P<0.05 *versus* pCO_2_: 40 mm Hg alone (control). Repeated four times

**Figure 6:**
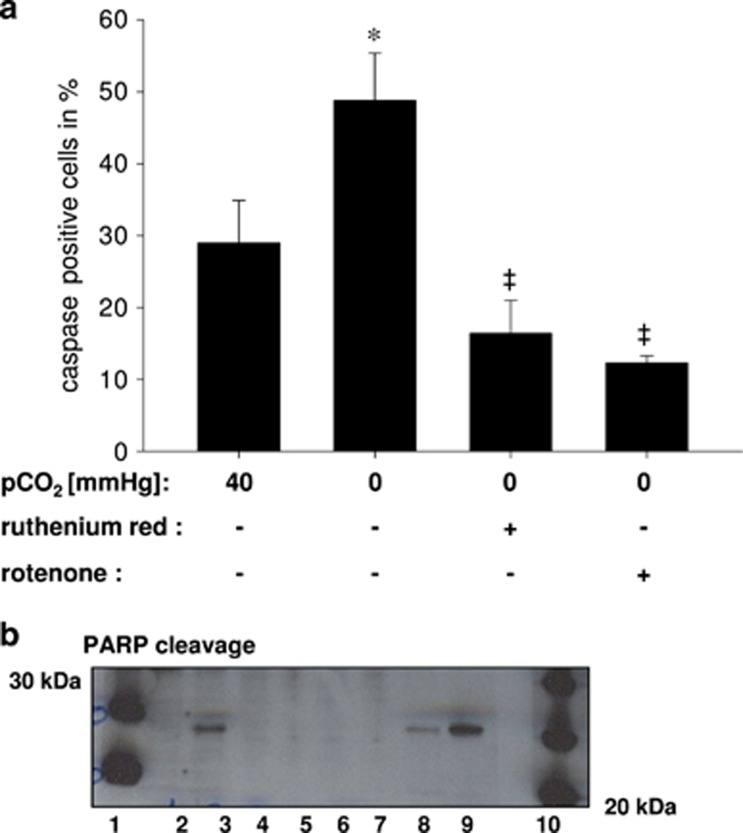
(**a**) Group data of caspase positive primary isolated AEC type 24 h following normocapnia (pCO_2_: 40 mm Hg), hypocapnia (pCO_2_: 0 mm Hg) alone or in combination with ruthenium red (10 *μ*M) or rotenone (1 *μ*M). Experiments were performed at constant extracellular pH of 7.4. Mean±S.E., ^#^*P*<0.05 *versus* baseline. *P<0.05 vs pCO_2_: 40 mm Hg alone (control), ^‡^*P*<0.05 *versus* pCO_2_: 0 mm Hg alone. Repeated four times. (**b**) Gels show PARP cleavage protein in native primary isolated AEC typ 2 (except lane 9). Lane 1 and 10: molecular weight ladder, lane 2: normocapnia, lane 3: hypocapnia, lane 4: normocapnia+rotenone, lane 5: normocapnia+ruthenium red, lane 6 hypocapnia+rotenone, lane 7: hypocapnia+ruthenium red, lane 8: staurosporine as positive control, lane 9: Hela cells+staurosporine as positive control

To investigate whether hypocapnia causes mitochondrial ROS production in primary isolated AEC type 2 cells we used the fluorescent MitoSOX Red mitochondrial superoxide indicator for live-cell imaging. We could show that under hypocapnia the ROS production, expressed as delta increase of the dye, was enhanced in comparison with normocapnia (Figure 5). To evaluate the respiratory chain as potential source of ROS we used rotenone under both normo- and hypercapnia. Rotenone almost diminished the hypocapnia-induced delta increase, whereas rontenone with normocapnia did not change ROS production in comparison with normocapnia alone (Figure 5). In addition, ROS production was also inhibited after ruthenium red incubation, which blocked the mitochondrial calcium uniporter and subsequently the mitochondrial Ca^2+^-uptake. Mitochondrial Ca^2+^ and ROS are potential trigger for apoptosis. Therefore, we used a CellEvent Caspase-3/7 fluorogenic substrate to detect apoptotic cells upon hypercapnia in a live-cell image setting. As depicted in Figure 6a, the percent of apoptotic cells increased from 20.1±5.9 under normocapnia to 48.8±6.6 under hypercapnia. Treatment with rotenone or ruthenium red prevented the initiation of apoptosis (23.8±8.1% apoptotic cells and 16.4±4.6% apoptotic cells, respectively) (Figure 6a). In order to validate these results we measured as an additional apoptosis marker the expression of cleaved poly-(ADP-ribose) polymerase (PARP) in primary isolated AECs type 2.Western blot analysis of this protein revealed that only cells cultured under hypocapnic conditions express cleaved PARP, which was inhibited by rotenone or ruthenium red (Figure 6b). Under normocapnic condition alone or in combination with rotenone or ruthenium red cleaved PARP was not detectable (Figure 6b).

## Discussion

**Figure 7:**
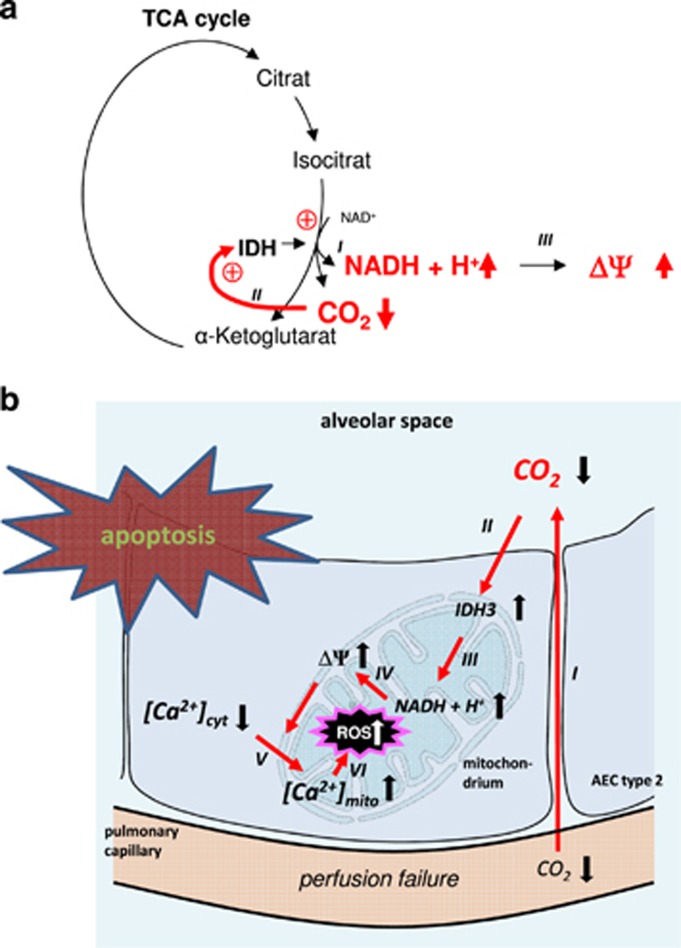
(**a**) IDH3 catalyzes the oxidative decarboxylation of isocitrate, producing *α*-ketoglutarate and CO_2_ while converting NAD^+^ to NADH (I). Because IDH3 activity is negatively regulated by its product CO_2_ we conclude that under hypocapnic conditions IDH3 activity (II) and thus NADH production (III) rises. (**b**) Proposed sequence of events underlying Ca^2+^-dependent CO_2_-sensing mechanism. Alveolar perfusion failure in ventilated lung regions, equivalent to a V_D_/V_T_ increase, leads to alveolar hypocapnia (I). Alveolar hypocapnia elevates IDH3 activity via a feedback mechanism (II). In turn, NADH (III) production and ΔΨ (IV) increases leading to a calcium shift from cytosol into the mitochondrium (V). Further, the hypocapnia-induced mitochondrial Ca^2+^ uptake leads to mitochondrial ROS production (VI). Finally, the mitochondrial Ca^2+^ uptake and/or ROS production results in apoptosis

We provide evidence that in AEC type 2 hypocapnia increases IDH3 activity and NADH production, ΔΨ, [Ca^2+^]_mito_, and mitochondrial ROS concentration, which in turn induces apoptosis (Figure 7).

For the first time, at least to our knowledge, we were able to measure an increase of the IDH3 activity in primary isolated AEC type 2 in response to hypocapnia. In this context, we have to critically emphasize that we could not see any increase of NADH concentration in AEC type 2 subjected solely to hypocapnia. Instead, after rotenone we could measure a steady rise of NADH concentration, which could further be increased in combination with hypocapnia. We concluded that NADH produced under hypocapnic conditions is immediately consumed by complex 1 and can thus not been detected. Inhibition of complex I however blocked the NADH consumption and after this intervention the hypocapnia-induced NADH production was detectable.

The dependency of the respiratory chain activity from NADH has been demonstrated by Kuznetsov *et al.*^19^ Hence, as a consequence of hypocapnia-induced elevation of NADH production, ΔΨ and thus the driving force for the mitochondrial Ca^2+^ uptake increased. This hypothesis was supported by the IDH3 knockdown. However, the IDH3 knockdown did not completely blocked the hypocapnia-induced NADH and ΔΨ responses. Reason for this result could be either that the IDH knockdown was not efficient enough or, more importantly, other mechanisms for the hypocapnia-induced NADH and ΔΨ responses are involved. Hypothetically, low pCO_2_ could also act on mitochondrial Ca^2+^ handling, e.g., on increase of mitochondrial Ca^2+^ influx or decrease of mitochondrial Ca^2+^ efflux. In this context another aspect has to be discussed: because IDH3 is one of the first enzymes of the TCA cycle and therefore upstream of all other TCA cycle enzymes, e.g., the CO_2_ producing alpha-ketoglutarate dehydrogenase, the IDH3 knockdown would affect these enzyme activities, which could also contribute to the hypocapnia-induced changes of in NADH and ΔΨ. This might be the reason why the IDH knockdown was highly efficient. In contrast, it could be assumed that the IDH3 knockdown would affect the overall flux through the TCA cycle. Unfortunately, we cannot exclude this hypothesis. However, we measured cytosolic Ca^2+^ responses to the purinergic receptor agonist ATP in IDH3-siRNA-treated AEC and the revealed Ca^2+^ responses were comparable to native and even to rotenone-treated AEC indicating that, at least, G-protein coupled receptor Ca^2+^ signaling is intact.

To determine whether the hypocapnia-induced increase of NADH and ΔΨ elicits a Ca^2+^ shift into mitochondria from the cytosol we measured both [Ca^2+^]_mito_ and [Ca^2+^]_cyt_. Accordingly, we could demonstrate on both primary isolated AEC type 2 and A549 cells that hypocapnia was accompanied not only by a [Ca^2+^]_mito_ increase but also by a [Ca^2+^]_cyt_ decrease. In order to differentiate whether the Ca^2+^ responses in AEC type 2 were CO_2_ rather than pH mediated, we additionally measured [Ca^2+^]_cyt_ primarily in A549 cells under several pH conditions and found that in AEC type 2 [Ca^2+^]_cyt_ response to CO_2_ is independent from pH.

Moreover, we could show that the hypocapnia-induced [Ca^2+^]_cyt_ response was inhibited by MCU knockdown or pharmacological blockade of the MCU with ruthenium red confirming a Ca^2+^ shift from the cytosol into mitochondria. From these experiments we further conclude that the driving force for the mitochondrial Ca^2+^ uptake came from the mitochondria and not from the cytosol since in response to hypocapnia [Ca^2+^]_cyt_ rather de- than increased. According to the literature, the [Ca^2+^]_mito_ increase can also be evoked by a Ca^2+^ shift from the ER directly into mitochondria. In our scenario this pathway seems to be unlikely because [Ca^2+^]_ER_ was unaffected by hypocapnia. However, we cannot exclude the possibility that the ER was synchronously re-filled with cytosolic Ca^2+^, whereas Ca^2+^ was shifted from the ER into mitochondria. It has also been described that mitochondria can uptake Ca^2+^ from the extracellular space. Indeed, this possibility, at least in part, cannot be excluded by our experiments, too.

The inhibition of the CO_2_-induced Ca^2+^ shift by rotenone and the unaffected [Ca^2+^]_ER_ suggests that the driving force for the mitochondrial Ca^2+^ uptake was an increase of the respiratory chain activity and hence ΔΨ rather than ER. This hypothesis was confirmed by our finding that the fluorescence of TMRM, which accumulates in mitochondria in a potential-dependent manner, was enhanced in both primary isolated AEC type 2 and in A549 cells under hypo- compared with normocapnic conditions. Addition of rotenone revealed, as expected, a quick decrease of the TMRM fluorescence indicating that the respiratory chain activity is the important determinant of ΔΨ. Further, hypocapnia induced an increase of the respiratory chain substrate NADH and the knockdown of the NADH-producing IDH3 inhibited NADH, ΔΨ, [Ca^2+^]_mito_, and [Ca^2+^]_cyto_ response under low CO_2_ levels.

Taken together, we suggested that CO_2_, a by-product of IDH3, inhibits IDH3 activity via a negative feedback loop. Under hypocapnic conditions, this inhibitory effect of CO_2_ on the IDH3 activity is reduced which subsequently increases NADH production and in turn ΔΨ which, at least in part, lead to a Ca^2+^ shift into mitochondria.

The hypocapnia-induced mitochondrial Ca^2+^ uptake was associated with AEC type 2 apoptosis. Ca^2+^ enters mitochondria via the Ca^2+^ uniporter. As its pharmacological inhibition by ruthenium red prevented both Ca^2+^ uptake and apoptosis we conclude that the hypocapnia-induced Ca^2+^ uptake was, at least in part, responsible for the apoptosis of AEC type 2. We can only speculate about the underlying mechanism. Increase in [Ca^2+^]_mito_ has been shown to induce apoptosis by alterations in mitochondrial membrane permeability, formation of the permeability transition pore, cytochrome c release, and activation of caspase-9 and -3 and disruption of ΔΨ. We could demonstrate that immediately after onset of hypocapnia ΔΨ increases. However, we have no data on ΔΨ 24 h after hypocapnia because TMRM measurements are useful only for a short time period. There are reports that especially Ca^2+^ coming from the ER might be one trigger for apoptosis. As stated above, this way of apoptosis induction might be possible in our scenario but we were not able to proof this hypothesis.

We could show that hypocapnia is associated with mitochondrial ROS production. As this response could be inhibited by rotenone we conclude that the hypocapnia-induced ROS was produced in the respiratory chain. In addition, the mitochondrial ROS production could be inhibited by ruthenium red. We therefore conclude that, at least in part, the hypocapnia-induced mitochondrial Ca^2+^ uptake was responsible for the ROS response. Although the Ca^2+^-dependent mitochondrial ROS production is well established we cannot give any proof for the mechanism of the Ca^2+^-dependent ROS production. Theoretically, it could be the case that the hypocapnia-induced increase of NADH production results in a higher amount of ubisemiquinone on complex III of the respiratory chain. This might statistically increases the probability that the electron will instead be donated to O_2_, generating superoxide. Furthermore, a reverse electron transport may occur under hypocapnia and be responsible for the hypocapnia-induced ROS production. Moreover, we cannot differentiate whether apoptosis was evoked directly by Ca^2+^, ROS, or both of them.

In summary, we identify a novel CO_2_-sensing mechanism of AEC type 2 in which the mitochondrial TCA enzyme IDH3 has a key role. Under hypocapnic conditions, IDH3 activity and thus NADH production increases, which subsequently induces mitochondrial Ca^2+^ uptake and ROS production leading to apoptosis. We propose that this mechanism plays an important role in the pathogenesis of ARDS because alveolar hypocapnia may occur in lung regions with high V_D_/V_T_. According to our results one could consider a novel ARDS therapy, namely CO_2_ inhalation in order to prevent hypocapnia-induced apoptosis of AECs and tissue injury in ARDS.

## Materials and methods

### Animals

Male Sprague-Dawley rats (350 g) were purchased from Charles River (Sulzfeld, Germany). After review and approval by the local ethics committee and the government of Hamburg, all animals were treated humanely in accordance with 'principles of laboratory animal care' (NIH Publication No. 86-23, revised 1985) as well as with the German legislation on protection of laboratory animals.

### Materials

Fura 2-AM, LysoTracker green (LTG), MTG, rhod-2, 2′, 7'-bis-(2-carboxylmethyl)-5-(and 6-) carboxyfluorescin-AM and TMRM were purchased from Molecular probes (Thermo Fisher Scientific, Waltham, MS, USA). Both rotenone and ruthenium red were purchased from Sigma-Aldrich (St. Louis, MO, USA). Vehicle for dyes and other agents was HBSS buffer (150 mmol/l Na^+^, 5 mmol/l K^+^, 1.0 mmol/l Ca^2+^, 1 mmol/l Mg^2+^, and 20 mmol/l HEPES at pH 7.4). All esterified fluorescent probes were prepared as stock solutions in dimethylsulfoxide.

### Primary AEC type 2 isolation and cell culture

Isolation and culture of AEC type 2 from adult male Sprague-Dawley rats according to procedures described previously.^20, 21^ In brief, after injection of 100 mg/kg ketamine and 20 mg/kg xylazinum (rompun), the lungs were surgically exposed and perfused with 3 × 10 ml F-12 K medium (ATCC, Manassas, VA, USA) supplemented with 25 mM HEPES (Sigma-Aldrich) to remove blood. After multiple lavages (5 × 10 ml) via the cannulated trachea with 5 mM ethylenediaminetetraacetic acid (EDTA, Sigma-Aldrich) and 5 mM ethylene glycol-bis (ß-aminoehtylether)-N,N’,N’-tetraacetic acid (Sigma-Aldrich) in a phosphate-buffered salt solution the lungs were removed from the body. Upon instillation of 20 ml warm F-12K medium (ATCC) enriched with 50 mM HEPES and 4.5 units/ml elastase Grade II (Worthington, Lakewood NJ, USA) the lungs were incubated at 37 °C for 30 min. After dissection of the large airways the lungs were added to 10 ml F-12K medium containing 25 mM HEPES, 20% fetal bovine serum (FBS, Biochrom AG, Berlin, Germany), and 100 *μ*g/ml DNAse (Typ IV, Sigma-Aldrich) quickly minced and incubated at 4 °C for 10 min. Then the solution was mixed by end-over end-rotation for 4 min and filtered successively through cotton gauze (1, 2, and 4 ply, respectively) following filtration through nylon mesh (100 and 40 *μ*m, BD Falcon, Corning, NY, USA). Upon centrifugation of the cell suspension at 150 × *g* for 10 min and resuspension in 20 ml warm DMEM (Biochrom AG) the cells were applied to plates covered with rat immunoglobulin (Ig) G (Sigma-Aldrich) for 1 h at 37 °C. The nonadherent cells were removed, collected in sterile tube, twice centrifuged at 150 × *g* for 10 min and resuspended in 3 ml of culture medium (DMEM with 10% FBS, 1% penicillin/streptomycin and 10 ng/ml keratinocyte growth factor (Sigma-Aldrich)). After determination of cell count and viability, cells were plated on collagen-treated coverslips and incubated at 37 °C and 5% CO_2_.

Human lung carcinoma A549 cells were grown in F-12K Medium (ATCC) with 10% FBS, 10 000 U/ml penicillin and 10 000 *μ*g/ml streptomycin (Biochrom AG). A549 is an alveolar epithelial cell line with type 2 cell characteristics including production of surfactant proteins. It has been widely used as a model to study the response of AEC in several conditions.^22, 23^

### Conventional fluorescent real-time cell imaging

For fluorescent imaging, a coverslip with fluorophore-loaded primary isolated AEC type 2 or A549 cells was mounted in an imaging/perfusion chamber on the stage of a conventional epifluorescence microscope (Olympus, Hamburg, Germany). The imaging chamber was perfused continuously with warm HEPES buffer (37 °C).

Fluorophores were excited by a mercury arc lamp illumination directed through appropriate interference filters and filter sets (Semrock, Rochester, NY, USA). Fluorophore exposure was controlled by a filter wheel (Sutter Lambda 10-C, Sutter Instrument Co). The fluorescence emission was collected using an objective lens (40 × water immersion, numerical aperture 0.8, Zeiss, Oberkochen, Germany) and captured with a charge-coupled device camera (Photometrics Coolsnap HQ^2^, Tuscan, AZ, USA).

*[Ca*^*2+*^]_*cyt*_ *determination.* For [Ca^2+^]_cyt_ measurements, cells were loaded for 45 min with 10 *μ*M fura 2-AM in physiological solution containing 0.02% pluronic. The cells were excited at 340 and 380 nm. The fluorescence emissions at 510 nm were recorded and [Ca^2+^]_cyt_ was calculated from a computer-generated 340:380 emissions ratio based on a dissociation constant of 224 nmol/l and appropriate calibration parameters.^24^

Fluorescence images were recorded under normo- or hypocapnic conditions in combination with an extracellular pH of 7.4 or 7.8 at 10 s intervals.

*[Ca*^*2+*^]_*mito*_ *determinations.* For [Ca^2+^]_mito_ measurements, cells were loaded with rhod-2-AM for 15 min followed by a Ringer's flush. To improve the specifity of [Ca^2+^]_mito_ measurement mitochondria were additionally localized by the fluorophore MTG, which binds specifically to the inner mitochondrial membrane.^25^ The wavelength for excitation was 560 nm, whereas monitoring the emission at 590 nm.

#### Mitochondrial membrane potential measurement

For mitochondrial membrane potential assessment, primary isolated AEC type 2 or A549 cells were loaded with TMRM. This indicator dye is a lipophilic cation accumulated by mitochondria in proportion to the electrical potential across their inner membrane (Δψ).^26^ The wavelength for excitation was 560 nm while monitoring the emission at 590 nm. Addition of the mitochondrial depolarizer FCCP (50 nM) resulted in a rapid loss of the mitochondrial TMRM fluorescence.

#### Mitochondrial NADH production

NADH concentration was quantified by measuring its auto-fluorescence intensity. Primary isolated AEC type 2 or A549 were excitated with 360 nm while monitoring the emission at 510 nm. For determining the mitochondrial production of NADH, its auto-fluorescence was co-localized with the mitochondria-specific marker MTG.

#### Determination of mitochondrial ROS Production

Mitochondrial ROS production was measured using MitoSOX Red (Thermo Fisher Scientific). Primary isolated AEC type 2 were stained with 10 *μ*M MitoSOX Red and 10 *μ*M MTG (staining of mitochondria) for 10 min at 37 °C. Upon washing with normocapia buffer, cells were then imaged with a conventional epifluorescence microscopy (Olympus) in normo- and hypocapnia puffer, respectively. Changes in the fluorescence intensity of MitoSOX Red were measured at 550 nm excitation and 600 nm emission wavelengths. In an additional set of experiments we preincubated the cell with 1 *μ*M rotenone to inhibit Complex I of the electron chain and/or 10 *μ*M ruthenium red to inhibit the MCU. As a positive control we used Antimycin AA (2 *μ*g/ml).

#### Determination of Caspase-3/7-positive cells

We used the Cell Event Caspase-3/7 Green Detection Reagent (Thermo Fisher Scientific) according to the manufacture’s instruction to determine apoptotic cells. In brief, upon incubation with normo- und hypocapnia puffer for 30 min at 37 °C primary isolated AEC type 2 were stained 24 h later with the caspase-3/7 reagent (10 *μ*M) and counterstained with the nuclei dye Hoechst 33342 (0.5 *μ*g/ml). Cells were then image with a conventional epifluorescence microscopy (Olympus) and caspase-3/7-positive cells were counted in four independent fields per well.

### 2-Photon and FLIM

To determine changes of NADH / NAD(P)H during inhibition of complex I and/or under hypocapnia treatment, FLIM was performed on a single-beam 2-photon microscope setup described elsewhere.^27^ In brief, the excitation wavelength of the pulsed Ti:Sa Laser (MaiTai, Spectra Physiscs) was 750 nm and sent through a water-dipping lens (20 ×, NA 0.95, WD 2 mm – Olympus) to the lung surface. Emission photons passed a band-pass filter (475/47 nm) and were finally detected at the Ultra-sensitive-port by a 16-channel time-correlated single photon counter, with a maximum rate of 78 MHz (LaVision Biotec, Bielefeld, Germany). Evaluation of the multi-exponential fluorescence signal was performed by the self-developed software RINIFLIM programmed in Matlab (MathWorks). For evaluation, Levenberg-Marquardt algorithms were used.

### IDH3 activity

The NAD^+^-dependent IDH3 activity was determined using the commercial available IDH activity assay kit according to the manufactures protocol (Sigma-Aldrich). In brief, the IDH3 activity in cell extracts was measured using the substrate isocitrate and the cofactor NAD^+^ in an enzyme reaction, which results in a colorimetric (450 nm) product proportional to NADH concentration. The enzyme activity was calculated according to the standard curve. One unit of IDH3 is the amount that will generate 1.0 *μ*mol NADH per minute at pH 8.0 at 37 °C.

### IDH3 and MCU knockdown

A549 cells were transfected with 10 nM stealth siRNA of IDH3 (Thermo Fisher Scientific) and/or 10 nM siRNA of MCU (Quiagen, Hilden Germany) and scrambled siRNA (Thermo Fisher Scientific and Quiagen) by using Lipofectamine RNAIMAX (Thermo Fisher Scientific) according to the manufacturer’s protocol. In brief, siRNA was diluted in 500 *μ*l Opti-MEM I medium (Quiagen) without serum in a well of the tissue culture plate. Lipofectamine RNAiMAX was added to each well containing the diluted siRNA. Cells were diluted in complete growth medium without antibodies so that the cell density after 24 h would be 30–50% confluent after plating.

### Real-time quantitative RT-PCR for IDH3 and MCU knockdown analysis

Total RNA was isolated using the RNeasy Kit (Quiagen). A total of 2 *μ*g of RNA were used for cDNA synthesis using the Maxima First Strand cDNA Synthesis Kit (Thermo Fischer Scientific) according to the manufacturer's instructions. qRT-PCR was performed with gene- and species-specific primers. cDNA template was amplified using the QuantiNova SYBR GreenPCR Kit (Quiagen) on a Rotor-Gene (Quiagen) according to the manufacture’s protocol. Quantitative analysis of data were performed by using the deltadeltaCT method.

### Western blot for IDH3 and MCU knockdown analysis

For immunoblotting, cells were washed twice with PBS and then scraped into RIPA buffer (Sigma-Aldrich) supplemented with the protease inhibitor cocktail complete Mini EDTA-free (Roche Diagnostics, Mannheim, Germany). Upon incubation on ice for 30 min lysed cells were centrifuged at 12 000 × *g* for 20 min (4 °C). The supernatants with the cytoplasmatic protein were collected, and protein concentrations were determined by using the bicinchoninic acid (BCA protein assay kit, Thermo Fisher Scientific), assay according to the manufacturer’s instructions. A total of 20 *μ*g samples were reduced in sample buffer (NuPAGE sample buffer, Thermo Fisher Scientific) plus reducing agent (NuPAGE sample reducing agent, Invitrogen) at 95 °C for 5 min and run on a 4–12% Bis-Tris polyacrylamid gel (NuPage, Thermo Fisher Scientific) and transferred to nitrocellulose filters (Thermo Fisher Scientific). The filters were blocked for 1 h at room temperature in blocking puffer (PBS plus 0.1% Tween 20 containing 5% milk powder and/or 5% bovine serum albumin (BSA)) with agitation. After application to the polyclonal rabbit IDH3A antibody (1:800, GenWay Biotech Inc., San Diego, CA, USA) or to the monoclonal rabbit MCU antibody (1:1000, Cell Signaling Technology, Danvers, MA, USA) overnight at 4 °C, the filters were exposed to an ECL horseradish peroxidise-conjugated donkey anti-rabbit IgG (1:10 000 and/or 1:20 000), GE Healthcare, Little Chalfont, UK) for 1 h at room temperature with agitation. After washing with washing puffer (PBS plus 0.1% Tween 20), the filters were developed with the ECL Plus Western Blotting Detection Reagents (GE Healthcare) according the manufacture’s protocol. For molecular weight standards, we used a MagicMark XP protein ladder (Thermo Fisher Scientific).

### Western blot analysis for PARP cleavage

Upon incubation with normo- und hypocapnia puffer for 3 h at 37 °C primary isolated AEC type 2 were collected 24 h later and prepared for immunoblotting as described in the section 'Western blot IDH3 and MCU knockdown analysis’. After gelelectrophoresis and immunoblotting, filter were blocked for 1 h at room temperature in blocking puffer (PBS plus 0.1% Tween 20 containing 5% BSA) with agitation. Upon application of the monoclonal rabbit Anti-cleaved-PARP antibody (1:500, Abcam, Cambridge, UK) overnight at 4 °C, the filters were exposed to an ECL horseradish peroxidise-conjugated goat anti-rabbit IgG (1:5000, Jackson ImmunlResearch Labaroties, Inc, West Grove, PA, USA) for 1 h at room temperature with agitation. After washing with washing puffer (PBS plus 0.1% Tween 20), the filters were developed with the ECL Plus Western Blotting Detection Reagents (GE Healthcare) according the manufacture’s protocol. For molecular weight standards, we used a MagicMark XP protein ladder (Thermo Fisher Scientific). As a positive control, we used staurosporine (1 *μ*M) treated cells.

### Statistics

All data are represented as means±S.E. Statistical data analysis was performed using SigmaStat (Systat Software, Chicago, IL, USA). Comparisons between two groups were tested using RankSumTest. Comparisons between more groups were tested by ANOVA on ranks followed by a pairwise multiple comparison. Repeated measurements were tested using the Mann–Whitney Rank Sum Test. Statistical significance was accepted at *P*<0.05.

## Publisher’s Note

Springer Nature remains neutral with regard to jurisdictional claims in published maps and institutional affiliations.

## References

[bib1] Kallet RH, Zhuo H, Liu KD, Calfee CS, Matthay MA. The association between physiologic dead-space fraction and mortality in subjects with ARDS enrolled in a prospective multi-center clinical trial. Respir Care 2014; 59: 1611–1618. 24381187 10.4187/respcare.02593 PMC4077995

[bib2] Laffey JG, Kavanagh BP. Hypocapnia. N Engl J Med 2002; 347: 43–53. 12097540 10.1056/NEJMra012457

[bib3] Laffey JG, Kavanagh BP. Carbon dioxide and the critically ill—too little of a good thing? Lancet 1999; 354: 1283–1286. 10520649 10.1016/S0140-6736(99)02388-0

[bib4] Shepard JW Jr., Hauer D, Miyai K, Moser KM. Lamellar body depletion in dogs undergoing pulmonary artery occlusion. J Clin Invest 1980; 66: 36–42. 6772668 10.1172/JCI109832 PMC371502

[bib5] Laffey JG, Engelberts D, Kavanagh BP. Injurious effects of hypocapnic alkalosis in the isolated lung. Am J Respir Crit Care Med 2000; 162: 399–405. 10934060 10.1164/ajrccm.162.2.9911026

[bib6] Myrianthefs PM, Briva A, Lecuona E, Dumasius V, Rutschman DH, Ridge KM et al. Hypocapnic but not metabolic alkalosis impairs alveolar fluid reabsorption. Am J Respir Crit Care Med 2005; 171: 1267–1271. 15764729 10.1164/rccm.200408-998OC PMC2718461

[bib7] Bachofen M, Weibel ER. Structural alterations of lung parenchyma in the adult respiratory distress syndrome. Clin Chest Med 1982; 3: 35–56. 7075161

[bib8] Lee KS, Choi YH, Kim YS, Baik SH, Oh YJ, Sheen SS et al. Evaluation of bronchoalveolar lavage fluid from ARDS patients with regard to apoptosis. Respir Med 2008; 102: 464–469. 17988850 10.1016/j.rmed.2007.10.001

[bib9] Tajeddine N. How do reactive oxygen species and calcium trigger mitochondrial membrane permeabilisation? Biochim Biophys Acta 2016; 1860: 1079–1088. 26922832 10.1016/j.bbagen.2016.02.013

[bib10] Chinopoulos C, dam-Vizi V. Mitochondrial Ca2+ sequestration and precipitation revisited. FEBS J 2010; 277: 3637–3651. 20659160 10.1111/j.1742-4658.2010.07755.x

[bib11] Starkov AA. The molecular identity of the mitochondrial Ca2+ sequestration system. FEBS J 2010; 277: 3652–3663. 20659159 10.1111/j.1742-4658.2010.07756.x PMC3725145

[bib12] Laraspata D, Gorgoglione V, La PG, Palmitessa V, Marzulli D, Lofrumento NE. Interaction of nitric oxide with the activity of cytosolic NADH/cytochrome c electron transport system. Arch Biochem Biophys 2009; 489: 99–109. 19653993 10.1016/j.abb.2009.07.020

[bib13] Brown GC. Control of respiration and ATP synthesis in mammalian mitochondria and cells. Biochem J 1992; 284: 1–13. 1599389 10.1042/bj2840001 PMC1132689

[bib14] Erecinska M, Wilson DF. Regulation of cellular energy metabolism. J Membr Biol 1982; 70: 1–14. 6226798 10.1007/BF01871584

[bib15] Erecinska M, Wilson DF, Nishiki K. Homeostatic regulation of cellular energy metabolism: experimental characterization *in vivo* and fit to a model. Am J Physiol 1978; 234: C82–C89. 204196 10.1152/ajpcell.1978.234.3.C82

[bib16] Blacker TS, Mann ZF, Gale JE, Ziegler M, Bain AJ, Szabadkai G et al. Separating NADH and NADPH fluorescence in live cells and tissues using FLIM. Nat Commun 2014; 5: 3936. 24874098 10.1038/ncomms4936 PMC4046109

[bib17] Niesner R, Peker B, Schlusche P, Gericke KH. Noniterative biexponential fluorescence lifetime imaging in the investigation of cellular metabolism by means of NAD(P)H autofluorescence. Chemphyschem 2004; 5: 1141–1149. 15446736 10.1002/cphc.200400066

[bib18] Palmer AE, Tsien RY. Measuring calcium signaling using genetically targetable fluorescent indicators. Nat Protoc 2006; 1: 1057–1065. 17406387 10.1038/nprot.2006.172

[bib19] Kuznetsov AV, Margreiter R, Amberger A, Saks V, Grimm M. Changes in mitochondrial redox state, membrane potential and calcium precede mitochondrial dysfunction in doxorubicin-induced cell death. Biochim Biophys Acta 2011; 1813: 1144–1152. 21406203 10.1016/j.bbamcr.2011.03.002

[bib20] Dobbs LG, Gonzales RFIsolation and culture of pulmonary alveolar Epithelial type II cells. In: Freshney RJ and Freshney MG (eds). Culture of Epithelial Cells, 2nd edn, Wiley-Liss, Inc, Hoboken: NJ, USA, 2002, pp 277–301.

[bib21] Qiao R, Yan W, Clavijo C, Mehrian-Shai R, Zhong Q, Kim KJ et al. Effects of KGF on alveolar epithelial cell transdifferentiation are mediated by JNK signaling. Am J Respir Cell Mol Biol 2008; 38: 239–246. 17872496 10.1165/rcmb.2007-0172OC PMC2214671

[bib22] Burvall K, Palmberg L, Larsson K. Metabolic activation of A549 human airway epithelial cells by organic dust: a study based on microphysiometry. Life Sci 2002; 71: 299–309. 12034348 10.1016/s0024-3205(02)01644-2

[bib23] Kwong KY, Literat A, Zhu NL, Huang HH, Li C, Jones CA et al. Expression of transforming growth factor beta (TGF-beta1) in human epithelial alveolar cells: a pro-inflammatory mediator independent pathway. Life Sci 2004; 74: 2941–2957. 15051419 10.1016/j.lfs.2003.08.048

[bib24] Kuebler WM, Ying X, Singh B, Issekutz AC, Bhattacharya J. Pressure is proinflammatory in lung venular capillaries. J Clin Invest 1999; 104: 495–502. 10449441 10.1172/JCI6872 PMC408527

[bib25] Poot M, Pierce RH. Detection of changes in mitochondrial function during apoptosis by simultaneous staining with multiple fluorescent dyes and correlated multiparameter flow cytometry. Cytometry 1999; 35: 311–317. 10213196 10.1002/(sici)1097-0320(19990401)35:4<311::aid-cyto3>3.3.co;2-5

[bib26] Scaduto RC Jr., Grotyohann LW. Measurement of mitochondrial membrane potential using fluorescent rhodamine derivatives. Biophys J 1999; 76: 469–477. 9876159 10.1016/S0006-3495(99)77214-0 PMC1302536

[bib27] Rinnenthal JL, Bornchen C, Radbruch H, Andresen V, Mossakowski A, Siffrin V et al. Parallelized TCSPC for dynamic intravital fluorescence lifetime imaging: quantifying neuronal dysfunction in neuroinflammation. PLoS ONE 2013; 8: e60100. 23613717 10.1371/journal.pone.0060100 PMC3629055

