# Supplementary material for: IDH3 mediates apoptosis of alveolar epithelial cells type 2 due to mitochondrial Ca2+ uptake during hypocapnia
Source: Cell Death Dis. 2017 Aug 24;8(8):e3005–. doi: 10.1038/cddis.2017.403 (PMC5596584; doi:10.1038/cddis.2017.403)
Supplement: Supplementary Information [file cddis2017403x1.docx]

**Supplement**

**IDH3 mediates apoptosis of alveolar epithelial cells type 2 due to mitochondrial Ca^2+^ uptake during hypocapnia.**

**Martina Kiefmann, PhD**^1^**; Sascha Tank, MD**^1^**; Paula Keller**^1^**; Christian Börnchen, PhD** ^1^**; Jan L. Rinnenthal, PhD**^2^**; Marc-Oliver Tritt**^1^**; Leonie Schulte-Uentrop, MD**^1^**; Cynthia Olotu, MD**^1^**; Alwin E. Goetz, MD, PhD**^1^**; Rainer Kiefmann, MD, PhD**^1^

^1^Department of Anesthesiology, University Hospital Hamburg-Eppendorf, Martinistrasse 52, 20251 Hamburg, Germany

^2^ Department of Neuropathology, Charite – Universitätsmedizin Charitéplatz 1 | Virchowweg 15, 10117 Berlin, Germany

*Corresponding author*:

Martina Kiefmann, PhD

University Hospital Hamburg-Eppendorf, Department of Anesthesiology,

Martinistrasse 52, 20251 Hamburg, Germany

phone: (49) 40 7410-57632, fax: (49) 40 7410-57631, e-mail: m.kiefmann@uke.de

Methods

**Transfection.** A549 cells were plated on chamber slides and grown overnight to a density of 80-90%. Transfection of 4mitD_3_CPV or D1ER plasmid DNA was performed using Fugene HD Transfection Reagent (Roche Diagnostics, Mannheim, Germany). Both FRET probes were a kind gift of Dr. Roger Tsien (University of San Diego, CA, USA). After 36-48 hours a transfection efficiency of 50-60% was reached and cells were subjected to Fluorescence Resonance Energy Transfer (FRET) measurement.

**Conventional fluorescent real-time cell imaging.** For FRET measurements emitted YFP and CFP fluorescence were captured simultaneously using a beamsplitter (Dual-View^TM^, Optical-Insights, New York, NY, USA) and an 100x water immersion objective (numerical aperture 1.0, Zeiss, Germany). Fluorescence was quantified using image analysis software (Metafluor, Molecular Devices, Sunnyvale, CA, USA).

*[Ca^2+^]_mito_ determinations.* [Ca^2+^]_mito_ measurements were performed on A549 cells using the FRET method. The FRET probe, 4mitD_3_CPV, consists of a mitochondrial located protein which changes its conformation when exposed to calcium. The change in protein conformation alters the distance between CFP and YFP which can be visualised as a change in CPF/YFP ratio. More details have been described elsewhere ^1^.

*[Ca^2+^]_er_ determination.* The FRET probe, D1ER, consists of an ER located protein which changes its conformation when exposed to calcium. The change in protein conformation alters the distance between CFP and YFP which can be visualised as a change in CPF/YFP ratio. More details have been described elsewhere ^1^.

*Cytosolic pH measurement.* A549 cells were loaded with the acetoxymethyl esters of BCECF (5 mM) for 30 min. The ﬂuorescence excitation ratio (495/438 nm) for each recording point was translated into intracellular pH using the high K1/nigericin *in vitro* calibration technique as described by Boyarsky and co-workers ^2^. Cytosolic pH was measured after induction of hypocapnia (0 mmHg pCO_2_).

**Biochemical determination of NADH concentration.** The NADH concentrations in cells were measured using the ultrasensitive EnzyChrom^TM^ NAD^+^/NADH Assay Kit from BioAssay Systems (Hayward, CA, USA). The assay based on a lactate dehydrogenase cycling reaction, in which the formed NADH reduces a formazan (MTT) reagent. The intensity of the reduced product, colour, measured at 565 nm, is proportionate to the NADH concentration in the sample. Briefly, the NADH was extracted from homogenized samples, heated at 60°C for 5 min, neutralized and then centrifuged at 14 000 rpm for 5 min. The NADH concentration in the supernatant were measured in a 96 plate with a microplate reader and calculated using a standard curve.

Literature

1. Palmer AE, Tsien RY. Measuring calcium signaling using genetically targetable fluorescent indicators. *NatProtoc* 2006, **1**(3)**:** 1057-1065.

2. Boyarsky G, Hanssen C, Clyne LA. Inadequacy of high K+/nigericin for calibrating BCECF. I. Estimating steady-state intracellular pH. *AmJ Physiol* 1996, **271**(4 Pt 1)**:** C1131-C1145.

Figure legends

**Figure 1 (A)** Images of primary isolated AEC type 2 show the pseudocolor-coded lifetime measured under normocapnic (pCO_2_: 40 mmHg) conditions. pH was at 7.4. **(B)** Quantification of τ_bound_ percentage distribution in primary isolated AEC type 2 under normocapnic (pCO_2_: 40mmHg) conditions. Mean±SE, repeated 4 times.

**Figure 2** Group data of NADH-concentration measured by a NADH-assay in native A549 under normocapnic conditions, hypocapnic conditions and after rotenone (1µM) under hypocapnic conditions at constant extracellular pH of 7.4. Mean±SE, * p<0.05 vs. normocapnia (control), ‡ p<0.05 vs. hypocapnia alone. Repeated 8 times.

**Figure 3 (A)** Images show the CFP or YFP fluorescence and the pseudocolor-coded CFP/YFP ratio of 4mtD3cpv transfected A549 cells under baseline condition (pCO_2_: 40 mmHg) and 5 minutes after switching to hypocapnic (pCO_2_: 0 mmHg) superfusion buffer. CFP and YFP fluorescence were simultaneously recorded using a beam splitter for the emitted light and a 100x objective. **(B)** Tracing of the mean CFP/YFP ratio from mitochondria of 4mtD3cpv transfected A549 cells superfused with normo- (pCO_2_: 40 mmHg) or hypocapnic (pCO_2_: 0 mmHg) buffer, as indicated. **(C)** Group data of maximal CFP/YFP ratio responses in 4mtD3cpv transfected A549 cells 5-10 minutes following switching from normocapnic (pCO_2_ of 40 mmHg) baseline to hypocapnic (pCO_2_: 0 mmHg) conditions or before (baseline conditions) or after ATP (10µM) or rotenone (1µM) application under normocapnic conditions. Experiments were performed at constant extracellular pH of 7.4 throughout. Mean±SE, # p<0.05 vs. baseline (pCO_2_: 40 mmHg). 2-3 cells were analyzed per picture. Experiments were repeated 3-5 times.

**Figure 4** Tracings of [Ca^2+^] from an identical A549 cell superfused with normo- (pCO_2_: 40 mmHg) or hypocapnic (pCO_2_: 0 mmHg) buffer, as indicated.

**Figure 5** Group data of the maximal [Ca^2+^]_cyt_-responses after ATP (10µM) in native A549 cells pretreated without or with rotenone or in A549 cells transfected with scrambled siRNA or IDH3-siRNA. Mean±SE, # p<0.05 vs. baseline (pCO_2_: 40 mmHg). 2-10 cells were analyzed per picture. Experiments were repeated 4-10 times

**Figure 6 (A)** Bars show group data of maximal [Ca^2+^]_cyt_-response in A549 cells 15 minutes after intervention. Under baseline conditions cells were superfused with buffer at following conditions: pCO_2_: 40mmHg and pH 7.4 for each group. Buffer conditions after intervention were as indicated. Mean±SE, # p<0.05 vs. baseline (pCO_2_: 40 mmHg), & p<0.05 vs. pCO_2_ of 40 mmHg at pH of 7.8. Repeated 8 times each. **(B)** Intracellular pH in A549 cells superfused with buffer at conditions as indicated. Mean±SE. 2-8 cells were analyzed per picture. Experiments were repeated 3 times.

**Figure 7 (A)** Images show the CFP or YFP fluorescence and the pseudocolor-coded CFP/YFP ratio of D1ER transfected A549 cells under normocapnic conditions (pCO_2_: 40 mmHg), 5 minutes after switching to hypocapnic conditions (pCO_2_: 0 mmHg), and 5 minutes after additional ATP (10µM) application. CFP and YFP fluorescences were simultaneously recorded using a beam splitter for the emitted light and a 100x objective. **(B)** Tracing of the mean CFP/YFP ratio of D1ER transfected A549 cells superfused with normo- (pCO_2_: 40 mmHg) or hypocapnic (pCO_2_: 0 mmHg) buffer and after additional ATP (10µM) application under hypocapnic conditions. **(C)** Group data of maximal CFP/YFP ratio responses in D1ER transfected A549 cells 5-10 minutes following switching from normocapnic (pCO_2_ of 40 mmHg) to hypocapnic (pCO_2_: 0 mmHg) conditions or 5 minutes after additional ATP (10µM) application under hypocapnic conditions. Experiments were performed at constant extracellular pH of 7.4 throughout. Mean±SE, # p<0.05 vs. baseline (pCO_2_: 40 mmHg). ‡ p < 0.05 vs. pCO_2_: 0 mmHg alone, 2-4 cells were analyzed per picture, Experiments were repeated 4 times.
