# Supplementary material for: IDH3 mediates apoptosis of alveolar epithelial cells type 2 due to mitochondrial Ca2+ uptake during hypocapnia
Source: Cell Death Dis. 2017 Aug 24;8(8):e3005–. doi: 10.1038/cddis.2017.403 (PMC5596584; doi:10.1038/cddis.2017.403)

Supplement Figure 1

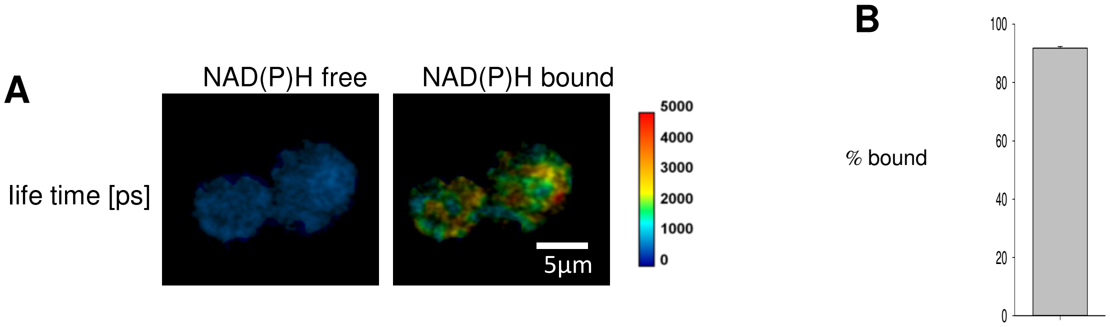

Supplement Figure 2

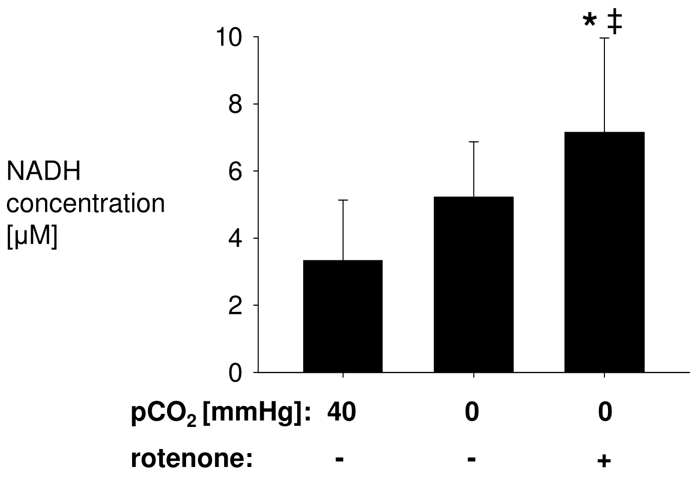

Supplement Figure 3

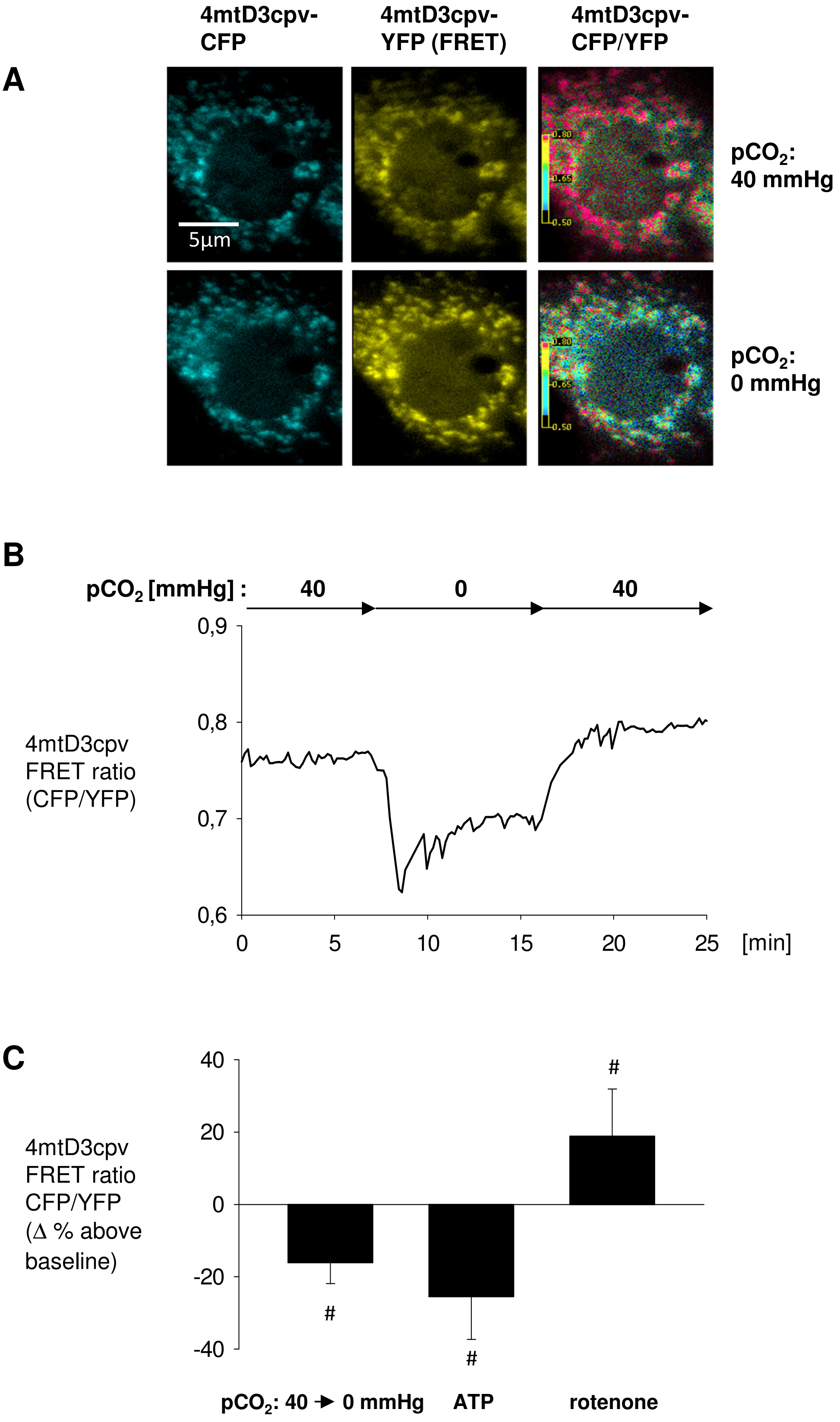

Supplement Figure 4

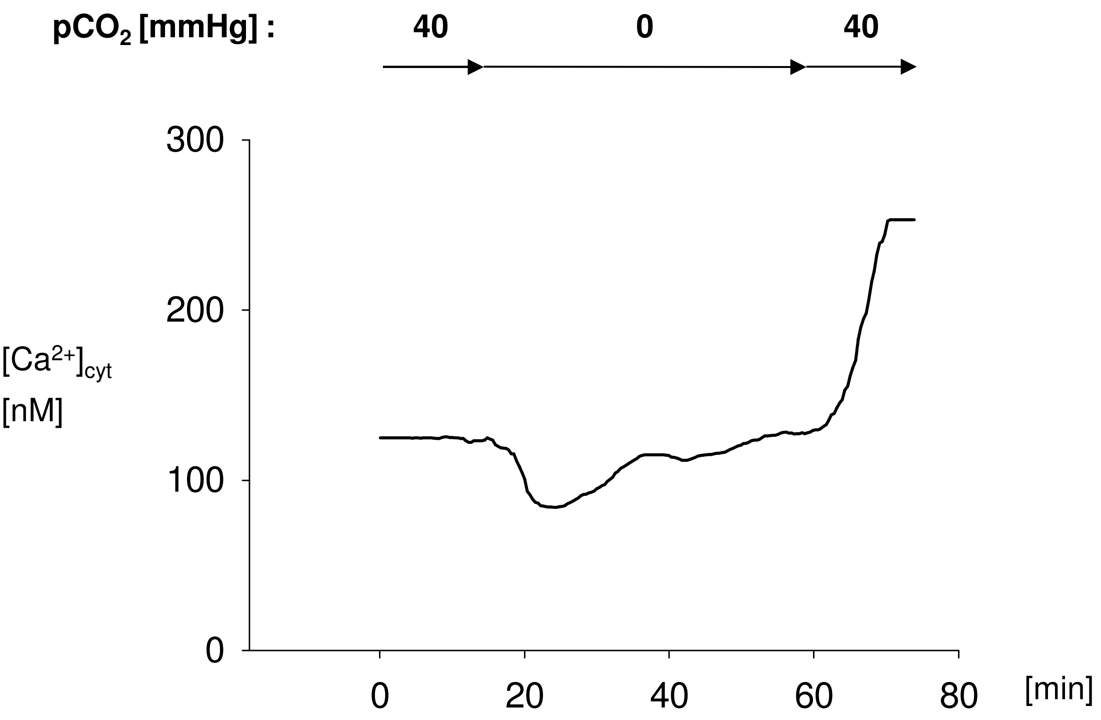

Supplement Figure 5

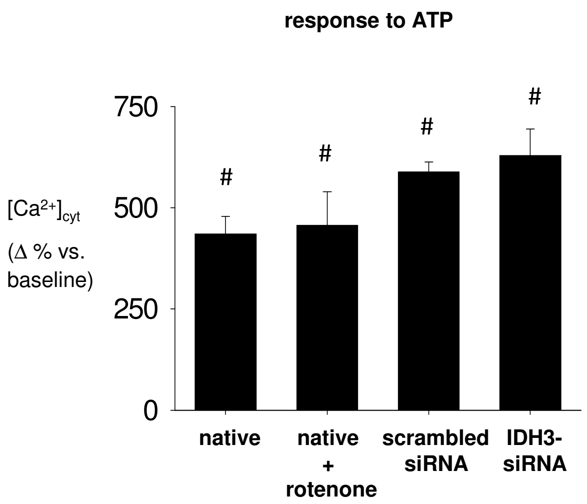

Supplement Figure 6

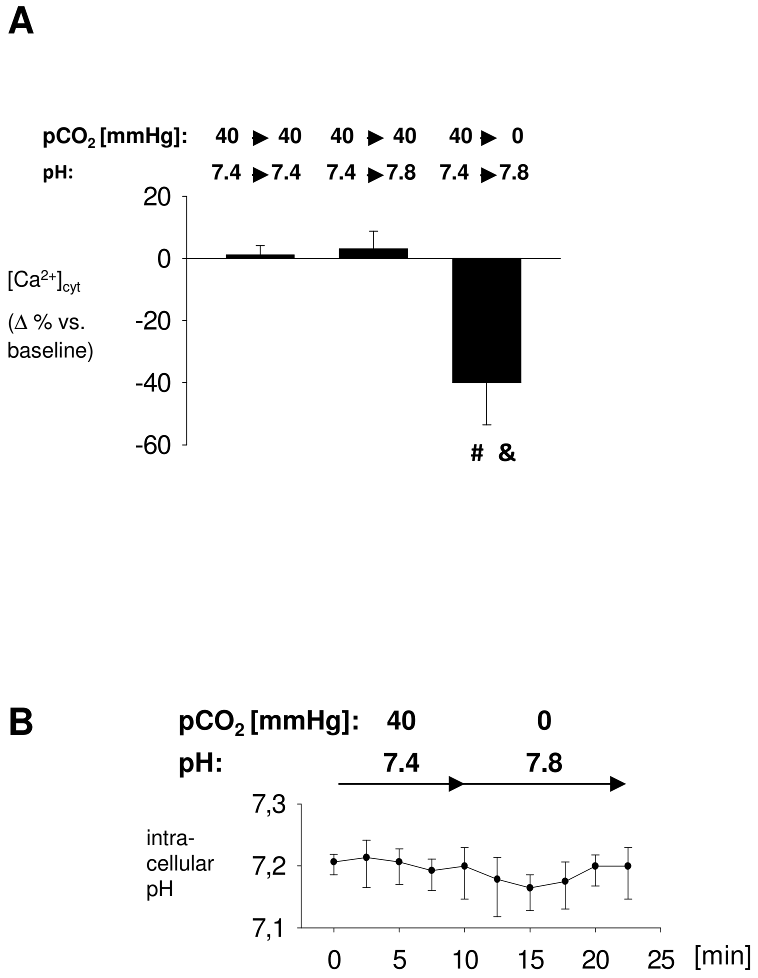

Supplement Figure 7

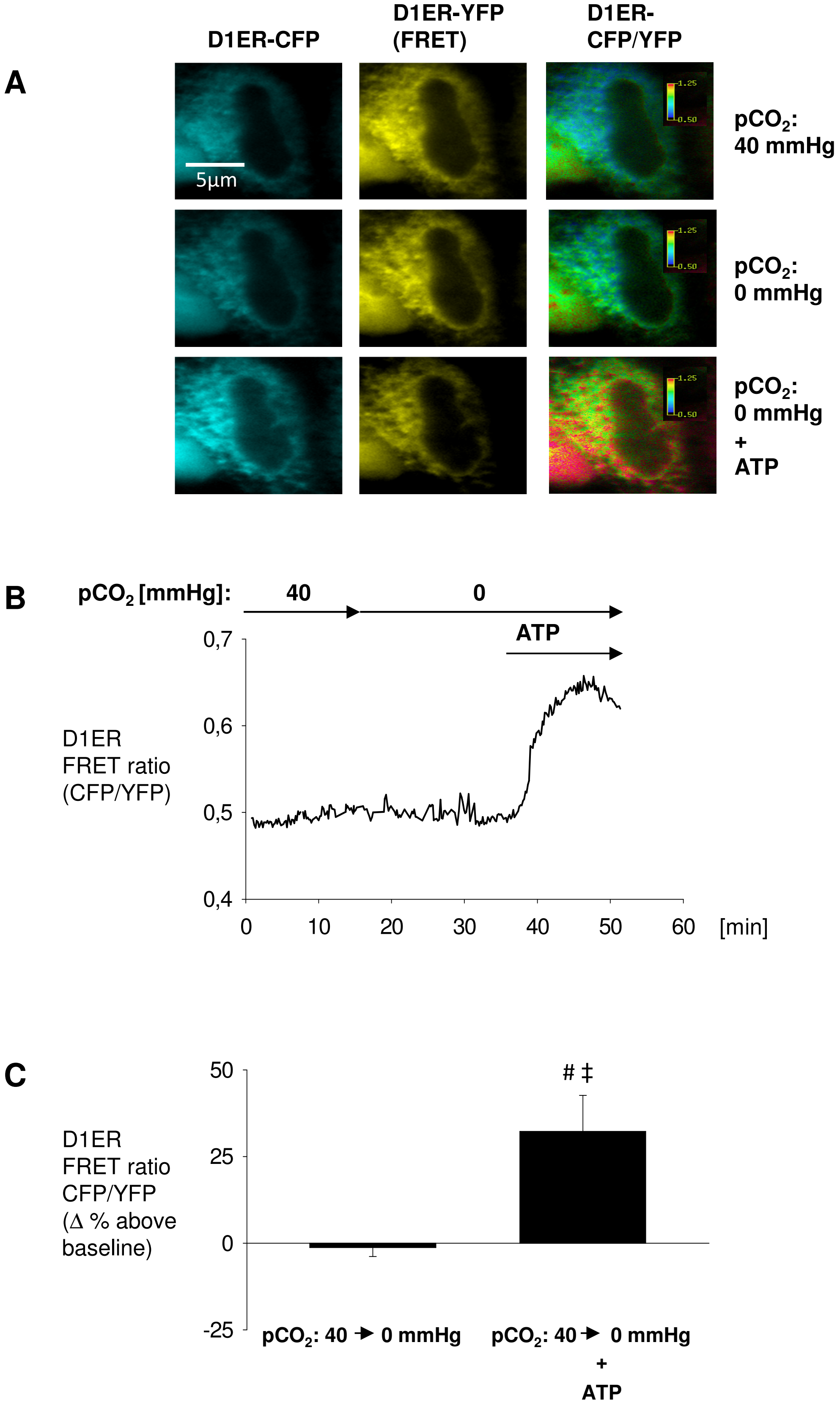

Supplement: Supplementary Figures [file cddis2017403x2.pdf]
